# Applications of Endovascular Brain–Computer Interface in Patients with Alzheimer’s Disease

**DOI:** 10.34133/research.1049

**Published:** 2025-12-23

**Authors:** Yuhao Sun, Danyang Chen, Qing Ye, Zheng You, Zhixian Zhao, Jian Shi, Haotong Sun, Shengjie Li, Xinran Xu, Yeguang Xu, Ping Zhang, Zhouping Tang

**Affiliations:** ^1^ Department of Neurology, Tongji Hospital, Tongji Medical College, Huazhong University of Science and Technology, Wuhan, Hubei, China.; ^2^ Big Data and Artificial Intelligence Office, Tongji Hospital, Tongji Medical College, Huazhong University of Science and Technology, Wuhan, Hubei, China.; ^3^ School of Mechanical Science and Engineering, Huazhong University of Science and Technology, Wuhan, Hubei, China.

## Abstract

Alzheimer’s disease (AD) is a prevalent neurodegenerative disorder affecting the elderly, leading to important impairments in cognitive function and the ability to live independently. This results in substantial disability and places an increasing burden on families and society. Currently, the therapeutic approaches adopted in clinical practice predominantly hinge upon cholinesterase inhibitors and the *N*-methyl-d-aspartate (NMDA) receptor antagonist memantine. Nevertheless, these medications merely alleviate symptoms and fail to tackle the pathological characteristics of AD. In recent years, monoclonal antibodies such as lecanemab and donanemab against β-amyloid (Aβ) have shown good efficacy in clinical practice for early-stage AD patients. However, the early diagnosis of AD remains a challenge. Against this backdrop, endovascular brain–computer interface (EBCI) offers an integrated solution for the early diagnosis and neuroregulatory treatment of AD patients, with minimal invasiveness. This review comprehensively examines the safety and feasibility of EBCI for AD patients, focusing on 3 major application areas: early diagnosis, deep brain stimulation targeting specific brain regions, such as the fornix and the basal nuclei of Meynert, and the use of external neurofeedback devices. Furthermore, we explore future development trends in this field, including miniaturization, integration, and the exploration of deep brain regions.

## Introduction

Alzheimer’s disease (AD) is a progressive neurodegenerative disorder and the leading cause of dementia in the elderly. It is characterized by the gradual onset of synaptic dysfunction, neuronal loss, and cognitive decline, typically resulting in a slow deterioration of the ability to live independently. This is accompanied by a decline in cognitive function and an increasing dependency on others. Early diagnosis of AD remains challenging, as both cognitive abilities and overall prognosis deteriorate with disease progression, often leading to diagnosis only in the later stages.

Currently, the primary pharmacological treatments for AD include cholinesterase inhibitors and the *N*-methyl-d-aspartate (NMDA) receptor antagonist memantine [1]. However, these treatments are associated with adverse effects such as hallucinations and headaches, and they offer only symptomatic relief, failing to halt the irreversible progression of the disease. Although the anti-β-amyloid (Aβ) monoclonal antibodies such as lecanemab and donanemab that have been clinically used in recent years have achieved good results in delaying the onset of AD by targeting the pathological mechanism of AD, these drugs are expensive, are not suitable for all patients, are not effective for all patients, and may have potential long-term side effects. The ideal intervention for AD should be the combination of early diagnosis and early treatment. Therefore, there is an urgent need for the development of alternative therapies that enable early diagnosis and address the underlying pathological features of the disease.

Brain–computer interface (BCI) technology represents a promising solution, providing an innovative method for direct interaction between the human brain and the external environment. This technology bypasses peripheral nerves, establishing a direct communication and control pathway, which allows for bidirectional transmission of information and commands between the brain and external devices. In this way, neural prostheses can be manipulated to restore motor function or stimulate specific brain regions to suppress disease manifestations, such as epilepsy [2]. BCIs are generally categorized into invasive, semi-invasive, and noninvasive types, depending on the depth of implantation, with signal strength typically decreasing across these categories. The endovascular brain–computer interface (EBCI) represents a subtype of invasive BCIs; however, it does not require a craniotomy. Instead, it leverages endovascular techniques to deliver electrodes into blood vessels near the target brain region, enabling neural signal acquisition and other functions. This approach offers signal acquisition performance comparable to that of subdural electrode array, but with substantially less implantation-related trauma than conventional invasive BCIs. By combining precise targeting of specific brain areas, high-fidelity signal acquisition, and minimal invasiveness, EBCI align well with the unique needs of patients with AD, who are particularly vulnerable to tissue damage and require region-specific interventions.

This review highlights the working principles and functional capabilities of EBCI and evaluates their feasibility and safety for the early diagnosis and treatment of AD.

### Materials and Methods

To conduct this systematic review, we searched Scopus, PubMed, IEEE, and ScienceDirect databases using the following strategy. No restriction was applied for publication year. Only studies meeting all of the following criteria were included: (“Endovascular Brain-Computer Interface” OR “Vascular Stent-Electrode” OR “Intravascular Neural Interface”) OR (“Alzheimer’s Disease” OR “AD” OR “Senile Dementia”) AND (“Neuromodulation” OR “Cognitive Enhancement” OR “Memory Improvement”).

The inclusion criteria were as follows: Peer-reviewed original research articles, including animal experiments, in vitro studies, clinical case reports, and cohort studies, as well as systematic reviews and meta-analyses that explicitly addressed at least one of the following topics: (a) the technical principles, biocompatibility, or signal acquisition/stimulation performance of EBCI or stentrode systems; (b) the rehabilitation or diagnostic applications of EBCI or stentrode in patients with AD; or (c) the prospects of EBCI or stentrode technology. When it was necessary to reference unpublished preclinical data due to the frontier nature of the field, such instances were clearly indicated in the text as “[not peer-reviewed; data cited from a conference abstract]”. For duplicate publications or those with excessive content overlap, only the most recent and data-complete version was retained.

In total, 347 relevant studies were initially identified using this search strategy. After screening titles and abstracts, 226 articles were excluded, and then 119 manuscripts and 2 extra reports were retained as the most relevant studies and were included in this review.

## Brain–Computer Interfaces

Traditional BCI systems function by recording neural signals generated in response to motor intentions, decoding these signals to convert them from neural to digital form, and utilizing neurofeedback to output digital signals that control neural prostheses. These systems can restore motor function or stimulate specific brain regions to suppress disease manifestations [2], such as in the case of epilepsy. Recent advancements in BCI research have demonstrated their remarkable motor control capabilities. For example, patients with quadriplegia have been able to type at speeds comparable to those of a standard smartphone keyboard [3] and control robotic prosthetics to perform tasks such as grasping and releasing objects, substantially improving their quality of life [4,5].

BCIs can be classified into 3 main categories: invasive, semi-invasive, and noninvasive. Invasive BCIs involve the implantation of electrode devices directly into the cortex to collect neural signals. Semi-invasive BCIs, such as electrocorticography (ECoG), are positioned within the skull but do not penetrate the cortex, and can be further categorized into epidural and subdural types. Noninvasive BCIs, by contrast, use external sensors, such as electroencephalography (EEG), magnetoencephalography (MEG), or near-infrared spectroscopy (NIRS), to capture neural signals without requiring brain implants. Generally, the degree of implantation injury follows this order: noninvasive < semi-invasive < invasive. However, signal quality typically follows the reverse trend, with noninvasive methods often yielding lower signal quality compared to invasive ones [6].

In recent years, BCIs have garnered substantial research attention, with rapid advancements in performance. Despite this progress, issues related to signal quality and stability remain persistent. Noninvasive BCIs face challenges due to signal dispersion resulting from the distance between the sensors and neural cells, as well as partial signal attenuation by the skull. This leads to weak signal strength and high levels of artifacts. On the other hand, invasive BCIs, especially those utilizing penetrating microelectrode arrays (MEAs), offer superior performance. They capture neural signals with high spatial and temporal resolution, providing an excellent signal-to-noise ratio and greater robustness against noise and motion artifacts. However, even in these systems, there is always some relative movement (<50 μm) between the electrodes and neurons [7], and fluctuations in electrical activity both inside and outside neurons can lead to unstable spike amplitudes. Additionally, electrode wear and the damage caused by invasive procedures can negatively affect signal stability [8].

Currently, MEA systems have been implanted in subjects for up to 5 years [9], but long-term implantation faces substantial challenges. Disruption of the blood–brain barrier (BBB) by MEAs triggers local inflammation and mediates neurotoxicity [10], which leads to neuronal degeneration and a gradual decline in recording performance. Increased BBB permeability allows the infiltration of local antigen-presenting cells, exacerbating inflammation [11]. This issue is especially pronounced with Michigan electrodes, which exhibit a faster rate of failure compared to other MEAs, likely due to higher BBB permeability and weaker wound healing responses. Although MEAs have advantages in promoting wound healing and reducing post-surgical BBB permeability [11], they still require frequent recalibration. Furthermore, the substantial damage caused by invasive MEA electrode arrays can induce reactive gliosis and scar formation [12], eventually resulting in the formation of a nonconductive barrier that isolates the electrodes from the target neurons [13]. This not only obstructs signal propagation but also increases impedance and extends the distance between the electrodes and the nearest target neurons.

While epidural and subdural electrodes reduce the risk of damage by being placed on the cortical surface, ECoG arrays positioned beneath the dura mater can only capture electrical activity from the cortical surface and are unable to measure signals from deeper cortical neurons. Moreover, their signal quality is generally inferior to that of fully invasive BCIs. In terms of hardware development, there has been a continuous effort to increase the number of channels in invasive BCIs to achieve higher signal resolution. However, simply increasing the number of channels in an attempt to improve data transmission rates and signal quality may not be a feasible solution in clinical settings, as the human brain cannot tolerate an unlimited number of invasive probes. In the signal acquisition process, a few high-quality probes can collect the vast majority of neural signals. Increasing the number of probes by several or even tens of times does not lead to more than a 10% improvement in signal acquisition efficiency. Therefore, indiscriminately increasing the number of channels is not a reasonable approach.

## Endovascular Brain–Computer Interface

**Fig. 1.**
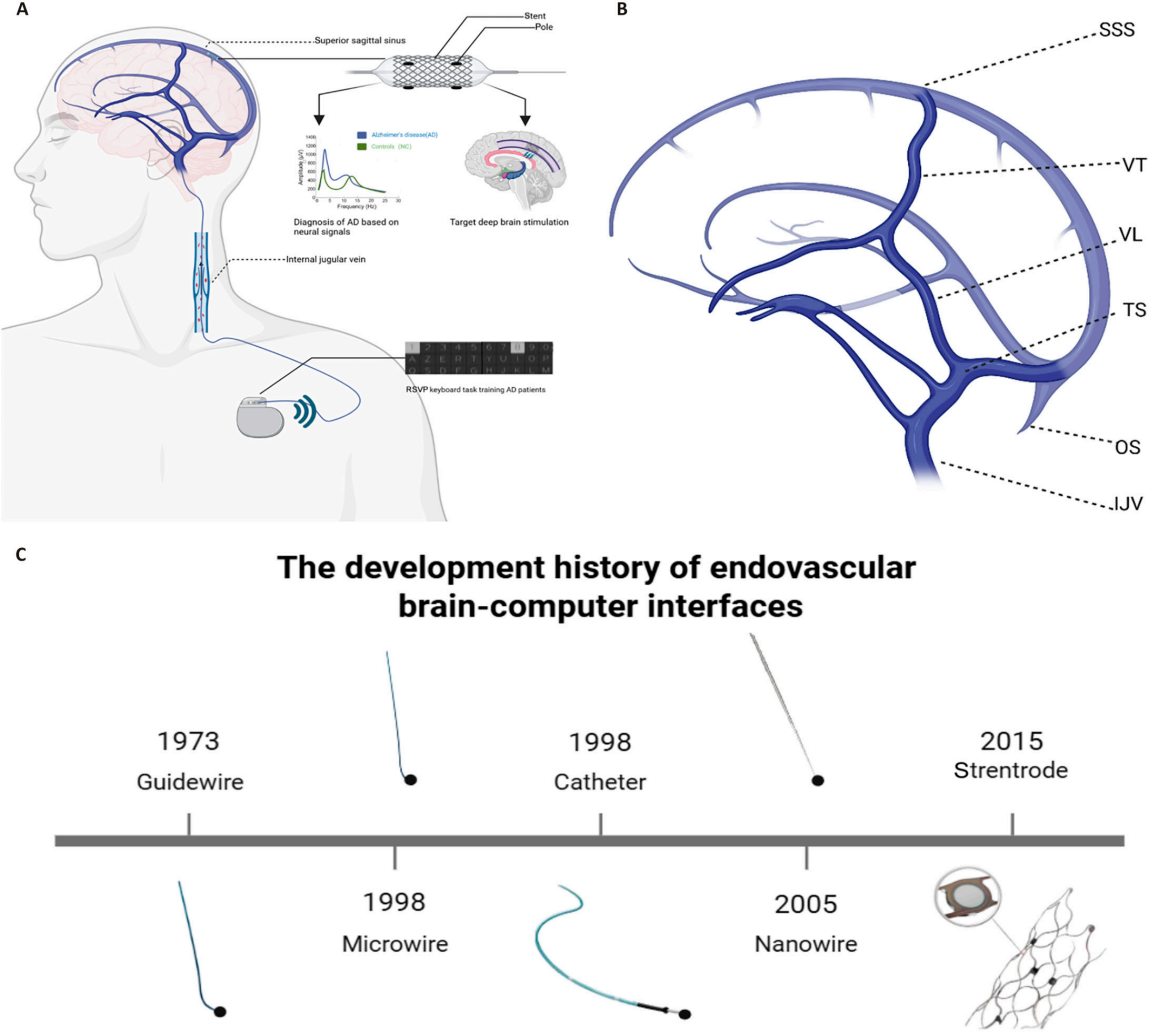
Schematic illustration of EBCI, including implantation sites and hardware evolution, created with BioRender. (A) Schematic diagram of an EBCI. The device is capable of both signal acquisition and DBS, and can be used in combination with other feedback hardware for attention training and neural circuit substitution, similar to other BCI systems. (B) Schematic representation of superficial cerebral veins. SSS, superior sagittal sinus; VT, vein of Trolard (superior anastomotic vein); VL, vein of Labbé (inferior anastomotic vein); TS, transverse sinus; OS, occipital sinus; IJV, internal jugular vein. (C) Hardware configurations at different developmental stages of EBCI: guidewire–microwire–catheter–nanowire–stentrode.

The EBCI is a promising technology classified as a specialized form of invasive BCIs. It offers stable and high-efficiency neural signal acquisition while minimizing physical trauma to the patient. Unlike invasive multi-electrode arrays (MEA) or subdural/epidural ECoG arrays, the EBCI does not require craniotomy. Instead, it utilizes vascular intervention to deliver electrodes to blood vessels near target brain regions for signal acquisition and other functions (Fig. 1A) [14]. This approach is particularly advantageous for elderly patients with poor prognoses for craniotomy, as it offers a less invasive alternative to traditional invasive BCIs. Compared to conventional invasive BCIs, EBCI offers stable and long-term access to deep brain regions—provided that appropriate adjacent vasculature exists—at a substantially reduced level of invasiveness. Unlike traditional invasive approaches, EBCI eliminate direct contact with neural tissue, thereby avoiding signal degradation caused by chronic inflammation-induced neuronal degeneration, as well as reducing the need for frequent recalibration due to signal instability. In comparison with semi-invasive BCIs such as ECoG, EBCIs demonstrate superior temporal and spatial resolution relative to epidural electrode arrays and slightly inferior performance compared to subdural arrays, as shown in Table 1. Moreover, EBCIs are capable of safely and efficiently recording and stimulating neural signals in deep brain structures.

**Table 1.** Comparison of EBCI with invasive and semi-invasive BCIs across dimensions such as spatial resolution and temporal resolution. Under the condition of having suitable target blood vessels, EBCI can provide high-quality signals in any brain region.

Index	EBCI	Invasive BCIs	Semi-invasive BCI
Signal type	LFP, spikes	Spikes, LFP	ECoG, LFP
Spatial resolution	Millimeter level (close to the cortex but limited by blood vessels, stronger than epidural BCI, slightly weaker than subdural BCI)	Sub-millimeter level (direct contact with neurons)	Sub-millimeter level (epidural BCI /subdural BCI)
Temporal resolution	Millisecond-level	Millisecond-level (the best)	Millisecond-level
Signal strength	2–5 times more powerful than EEG and is similar to ECoG	The most powerful modality, as it directly captures neuronal spiking activity	Moderate
Target brain areas	Virtually any brain region, as long as there is appropriately located vasculature nearby	Any brain region (the target area for implantation)	The cortical surface

Owing to their endovascular delivery, EBCI can access brain regions that are otherwise unreachable using conventional methods, including the medial temporal lobe and basal ganglia. These areas are particularly valuable in cases where cortical damage leads to signal loss. Beyond minimizing tissue trauma, endovascular electrodes also offer several operational advantages over subdural electrode arrays. For example, electrode array malfunction is a common issue in long-term neural recordings, and subdural arrays exhibit substantially higher dislodgement rates compared to endovascular electrodes. The larger degree of movement permitted in the subdural space—prior to anchoring to the dura mater and skull—increases the risk of electrode detachment or breakage. In contrast, endovascular electrodes, which are fixed within the vascular structure, greatly reduce the likelihood of such mechanical failures [15].

The first intracranial electroencephalogram (EEG) recorded from an endovascular approach was in 1973 when Penn et al. [16] improved upon the flexible vascular guidewire developed by Driller et al. [16,17], connecting it to a platinum–cobalt magnet used as the electrode.

As early as 2016, Bower et al. [18] demonstrated that microelectrodes could record cortical signals within blood vessels. Using a pig model, they introduced a MEA (40 μm in diameter) into the superior sagittal sinus (SSS) through a venous incision to measure cortical high-amplitude spikes induced by penicillin injection. They compared the results with subdural arrays, revealing that the spatial resolution of signals obtained from the EBCI was slightly inferior to subdural arrays but comparable to epidural arrays. This study confirmed that endovascular arrays could record neural signals with high accuracy and localize epileptic foci. The inferior signal quality from the endovascular interface, compared to subdural arrays, was primarily attributed to signal loss caused by the varying conduction distances in cerebrospinal fluid (CSF), rather than the dura mater (since the venous sinus is located above the dura).

Subsequent studies by He et al. [19] utilized guidewire electrodes to measure auditory and visual evoked potentials in pig models, yielding similar conclusions. However, the studies using guidewire electrodes were limited by the short duration of signal recordings, typically lasting only a few hours. This limitation poses a substantial drawback for the device’s potential future use in closed-loop control and long-term monitoring.

Opie and colleagues [20] made a breakthrough by demonstrating a stent-based device capable of long-term implantation in blood vessels to record neural activity. In their sheep model, they recorded cortical activity for up to 190 d using angiography and compared the data with subdural and epidural arrays. Their findings corroborated previous research, revealing that endovascular electrodes can record neural activity with similar quality to subdural arrays. Additionally, the electrochemical impedance spectrum of the device showed that peak resistance frequency stabilized after approximately 8 d, with more than 85% of the implanted stent pillars being covered by neointima within 2 weeks. This indicated that the stent electrodes could maintain long-term stability and recording capabilities within blood vessels [21]. Forsyth et al. [22] further validated the device’s long-term decoding ability in motor experiments [not peer-reviewed; data cited from 2019 IEEE/EMBS Neural Engineering Conference] (Table 2).

**Table 2.** Hardware characteristics of EBCI at different developmental stages, including dimensions and commonly used materials

Stage	Dimension range	Typical materials
Guidewire	Diameter 100–300 μm	Stainless steel, nitinol alloy
Microwire	Diameter 20–50 μm	Platinum–iridium alloy, tungsten wire, and nichrome wire
Catheter-based	Outer diameter 200–500 μm	Polyurethane/silicone tubing with embedded gold/platinum electrodes
Nanowire-based	Electrode size tens to hundreds of nanometers; membrane thickness <10 μm	Carbon nanotubes, silver/gold nanowires, graphene–PI (polyimide) thin films
Stentrode	Stent outer diameter 2–4 mm; wire diameter 50–100 μm	Nitinol or cobalt–chromium stent framework with platinum/gold electrodes

As mentioned above, advancements in miniaturization and the need for long-term recordings have led to the evolution of endovascular recording devices from guidewire recordings to microfilament/nanowire recordings, and then to catheter- and stent-based recordings (Fig. 1C). The most advanced BCIs currently combine a stent with an electrode array. Before implantation, cyclic voltammetry (CV) was conducted to clean residual substances from the electrode surface, thereby reducing foreign body reactions and lowering electrode impedance (from 6.26 ± 6.7 kΩ to 2.21 ± 1.2 kΩ) to enhance signal stability. The Pt–W signal transmission lines were insulated with polyimide (PI) to prevent electrical interaction with blood or surrounding tissues during signal transmission [23] [not peer-reviewed; data cited from 2024 International Winter Conference on BCI], further improving the stability of the electrode array. The self-expanding stent securely holds the electrode array in place, facilitating prolonged signal acquisition and therapeutic use. In the past years, EBCIs have shown their innovative potential. In March 2025, Synchron announced a collaboration with Nvidia on its latest endovascular BCI (stentrode), which integrates Nvidia’s Holoscan platform with Apple Vision Pro to achieve faster and more precise neural signal decoding and interaction [24] [not peer-reviewed; data cited from Wired magazine technology news article]. The system is equipped with an embedded artificial intelligence (AI) that not only decodes brain signals but also leverages large-scale neural data to train foundation models, advancing from supervised learning to more intelligent self-supervised learning. This AI model, known as Chiral, was designed to learn the “language of human cognition”. Rather than merely recognizing signals, it interprets user intentions and adapts its operations dynamically within specific contexts, potentially enabling more complex and natural interactions in the future.

At the NVIDIA GTC conference, a compelling demonstration was given by a patient with amyotrophic lateral sclerosis (ALS), Rodney Gorham, who directly controlled smart home devices. For instance, he played music, adjusted lights, turned on a fan, fed a pet, and operated a robotic vacuum via a visual interface, all without physical movement or speech. Synchron previously conducted an implantation in a 65-year-old ALS patient, Mark Jackson, in 2023. In a 2025 demonstration, he showcased the ability to use the implanted BCI device to perform tasks, such as gaming, messaging, and online shopping. His performance was remarkably accurate, achieving 13 of 14 successful target hits, reaching 100% accuracy [25] [not peer-reviewed; data cited from Wired magazine technology news article].

The EBCI has already been successfully implanted in patients with limb paralysis for up to 12 months [14]. In a pioneering human clinical trial, participants with upper limb paralysis who received an EBCI were able to independently operate a laptop for various daily activities. The device was implanted in 4 male participants in Australia, all of whom had severe bilateral upper limb paralysis due to ALS or primary lateral sclerosis (PLS) [14]. Participants underwent dual antiplatelet therapy for 3 months and continued aspirin treatment for 12 months to prevent clotting. A year after surgery, the implanted BCIs remained securely positioned in the sagittal sinus, with none of the participants experiencing prolonged neurological effects or severe adverse events, such as vascular occlusion or thrombosis. Throughout the study, the BCIs maintained stable signal strength, enabling the participants to successfully control a computer to send emails, text, engage in online banking, and shop.

Moreover, since the device is located within blood vessels and does not directly contact neural tissue, targeted stimulation can be achieved with minimal neural damage. Elderly patients often exhibit low tolerance for craniotomy, which limits the use of invasive methods and hinders the application of deep brain stimulation (DBS) techniques, which are beneficial for AD (NCT03622905 and NCT01094145). The clinical efficacy of DBS on patients with AD will be elaborated in detail in the “The application of DBS in the treatment of AD patients” section. The application of DBS in the treatment of patients with AD is discussed in this article.

In contrast, the EBCI provides a safe and effective therapeutic approach, making it particularly important in the treatment and early diagnosis of AD patients.

## AD Patients as the Target for Application

AD is characterized as a network disconnection disorder, with impaired synaptic plasticity being a key hallmark. Intact synaptic plasticity plays a crucial role in neuronal oscillations and is considered essential for information transmission at the cellular level, which is vital for cognitive function. Future treatments for AD can generally be divided into 2 primary directions [26]: one involves targeting and clearing biochemical markers, such as neurofibrillary tangles [composed of phosphorylated tau (p-tau)] and amyloid plaques (Aβ), to prevent or slow neurodegeneration [27]; the other aims at enhancing neuronal connectivity to improve cognitive function [28]. Both approaches leverage the potential of BCIs. Theoretically, stimulation of specific brain regions via electrode arrays may reduce Aβ deposition in patients with AD by modulating pathological nonlinear connectivity patterns, thereby improving cognitive function. Clinical studies have further shown that DBS exerts beneficial effects through multiple mechanisms, including the up-regulation of endogenous neurotrophic factors, enhanced synaptophysin expression, and promotion of neurogenesis. These effects collectively contribute to a slowing of disease progression and an improvement in cognitive performance in AD patients. A detailed discussion of these mechanisms will be provided in subsequent sections of this article. However, for elderly individuals, a group at high risk for AD, noninvasive BCIs are challenging to wear long-term and may not provide effective stimulation due to issues like scalp oils and sweat. Conversely, traditional invasive BCIs cause substantial tissue damage, raising safety concerns. Research has shown that chronic inflammation and brain tissue damage resulting from invasive BCIs within the first 6 weeks are independent of device size and cannot be mitigated by reducing the device volume [29]. This problem is even more pronounced in the elderly, whose postoperative frailty—due to factors such as hypertension, diabetes, and infections—can negatively impact craniotomy outcomes [30,31]. Age itself is also a strong predictor of poor outcomes following craniotomy [32]. Compared to traditional open-skull BCI implantation, endovascular approaches offer the advantages of minimal invasiveness, shorter recovery time, and enhanced safety—features that align well with the specific clinical needs of AD patients.

### Implantation sites and safety of EBCI in AD patients

The implantation procedures for the EBCI are well-established in terms of safety. Currently, most procedures target the brain’s venous sinuses. Venous implantation substantially reduces the risk of thrombosis compared to arterial implantation, and it allows access to regions near the primary and secondary sensory-motor cortices [33], facilitating the decoding of motor intent and providing neural feedback. The venous system is closely connected to the subdural space and cortical areas, making it a focal point for future research. Among the venous structures, the Trolard vein (anastomotic superior vein) and the Rolandic vein (central sulcus vein) are of particular interest [34]. Notably, the Trolard vein is present in 26% to 80% of patients, with the most common location being the cortical area posterior to the central sulcus, near the sensory centers, providing abundant signal sources (Fig. 1B). The Trolard vein is the largest cortical vein draining into the SSS, with an average diameter ranging from 2.14 to 3.32 mm. In the current sheep model, the smallest lumen diameter of a catheter that can safely deliver to the SSS is 1.1 mm [33], making it a feasible option for implantation.

Regarding the safety of cerebral venous sinus or cerebral venous stent implantation, surgical complications such as intracranial hemorrhage and thrombosis must be carefully considered. To mitigate the risk of stent-induced thrombosis after implantation, current human studies employ anticoagulation strategies, including dual antiplatelet therapy for 3 months post-implantation, followed by a 12-month course of aspirin monotherapy as a preventive measure. During follow-up, no thrombotic events were reported among the implanted subjects [14,35]. In a similar context, Ahmed et al. [36] reported outcomes from cerebral venous sinus stent implantation, noting that while the sample size was small (52 patients), the thrombosis rate was 0%. In a systematic review of stent implantation by Townsend et al. [37], although no specific thrombosis incidence was mentioned, 2 cases were highlighted. One case involved a postpartum woman in her 20s who developed intrastent thrombosis after anticoagulation therapy, and the other involved delayed thrombosis. Both patients achieved excellent recovery, and the researchers attributed thrombosis formation to a poor response to anticoagulants in these particular patients [37,38]. Notably, following a thrombotic event, the venous blood flow toward the heart can impede the effective administration of thrombolytic agents, making thrombus recanalization more challenging. Using contralateral collateral circulation for thrombolysis could present a promising strategy.

As for the risk of intracranial hemorrhage following anticoagulation, a meta-analysis by Nicholson et al. reported a 3% incidence of major postoperative complications (including intracranial hemorrhage) in this procedure. These complications included acute subdural hematomas (SDHs) and microcatheter-induced subdural punctures, all of which had excellent recovery post-bleeding. Townsend et al. [37] suggested that a substantial proportion of complications could be attributed to intraoperative factors. They provided key technical management guidelines for venous sinus stent implantation, such as the possibility of intracranial hemorrhage caused by inadvertent guidewire puncture during access to the venous sinus. Using a softer guidewire could help further reduce hemorrhage rates.

It is important to acknowledge that AD and cerebral amyloid angiopathy (CAA) share a common pathological hallmark—Aβ deposition. Given that CAA is a major risk factor for intracranial hemorrhage, it is crucial to carefully evaluate the risks associated with electrode implantation in AD patients who also present with CAA. The 2016 European Society of Cardiology guidelines for atrial fibrillation recommend avoiding anticoagulant use in patients with CAA [39]. However, a 2018 cohort study found that the incidence of symptomatic intracranial hemorrhagic events in patients with a history of atrial fibrillation, stroke, and cerebral microbleeds was only 9.8/1000 patient-years [40]. Moreover, the substitution of high-risk anticoagulants like warfarin with medications such as clopidogrel has been shown to further reduce the risk of bleeding.

Additionally, the future target population for EBCI in AD will primarily consist of patients with mild AD, who typically exhibit less severe cerebral amyloid changes. The risk of bleeding caused by this will also be greatly reduced. Considering this, and as discussed earlier, the current technique for cerebral venous sinus stent implantation holds substantial potential for improvement. Therefore, it is likely that further advancements will enhance the safety of this procedure in the treatment of AD.

### The potential of EBCI in the early diagnosis of AD patients

Early detection is critically important in neurological disorders, particularly for conditions such as stroke [41] and AD, where early identification and timely intervention can yield substantial benefits: AD is a progressive neurodegenerative condition with subtle early symptoms, making early diagnosis a substantial challenge. By the time the disease becomes evident, it is often in its mid-to-late stages, where treatment options become less effective. A substantial body of literature indicates that most therapeutic interventions are targeted at patients with mild AD, as the irreversible brain damage in severe AD patients is often too extensive, and their weak post-treatment feedback further hinders research development [42–44]. Therefore, early detection of AD is crucial for achieving effective outcomes with other therapies.

Currently, AD diagnosis in clinical practice largely relies on medical history, clinical symptoms, mental rating scales, and structural magnetic resonance imaging (MRI) to detect hippocampal atrophy. However, this diagnostic approach is not entirely reliable. In younger patients with atypical symptoms or without the “typical” hippocampal volume loss, AD may go unrecognized. In a neuropathologically confirmed cohort of early-onset AD, 37.5% of patients exhibited non-memory-related symptoms, with behavioral and executive function deficits being the most common atypical presentations. Among patients with atypical symptoms, 53% had incorrect initial clinical diagnoses [45,46].

Fortunately, the development of in vivo biomarkers has substantially advanced the diagnosis of AD, shifting detection from the late-stage dementia phase to earlier stages and offering the potential for pre-symptomatic diagnosis. Positron emission tomography (PET) imaging, using radiolabeled tracers specific to pathological biomarkers, enables in vivo molecular imaging to provide quantitative and visualized biological information. Clinically, PET imaging agents targeting amyloid proteins can examine amyloid deposition in the brain. After the injection of a tracer targeting amyloid, the imaging agent binds specifically to amyloid proteins due to its conjugated aromatic ring structure, which allows precise binding. In both typical and atypical AD cases, amyloid deposition exhibits similar characteristics, occurring diffusely throughout the neocortex, with early involvement of the posterior medial cortex. The medial temporal lobe, primary sensory motor, and visual cortices are relatively spared [47]. Based on this principle, PET-Tau and other imaging methods can also differentiate between typical and atypical AD presentations. The development of in vivo biomarker-based diagnostics is also influencing international guidelines for the diagnosis of AD. The clinical diagnostic criteria for AD are gradually shifting from a focus on clinical manifestations alone toward an emphasis on defining AD based on a combination of biological markers and clinical presentation (Table 3).

**Table 3.** International diagnostic criteria for AD increasingly emphasize a combined assessment of clinical manifestations and biomarkers, reflecting the advancement of biomarker-based diagnostic technologies

Standard	Year	Scope	Key features
NIA-AA 2011 (National Institute on Aging—Alzheimer’s Association)	2011	Clinical and research	Alzheimer’s disease (AD) is divided into 3 stages: 1. Preclinical: asymptomatic but with abnormal biomarkers. 2. Mild cognitive impairment due to AD: mild impairment of memory or other cognitive function, with no substantial impact on daily life. 3. Dementia due to AD: substantial cognitive decline, affecting daily life.
DSM-5-TR (Diagnostic and Statistical Manual of Mental Disorders, Fifth Edition, Text Revision)	2022	Clinical	Cognitive decline must affect daily life and other causes must be excluded. Genetic testing or imaging can be used to confirm the diagnosis.
NIA-AA 2024 Revision (Research Framework)	2024	Research	Emphasize the diagnosis of AD based on biological markers rather than relying solely on clinical symptoms. View AD as a continuous pathological process. Biological markers play a critical role in diagnosis.

**Fig. 2.**
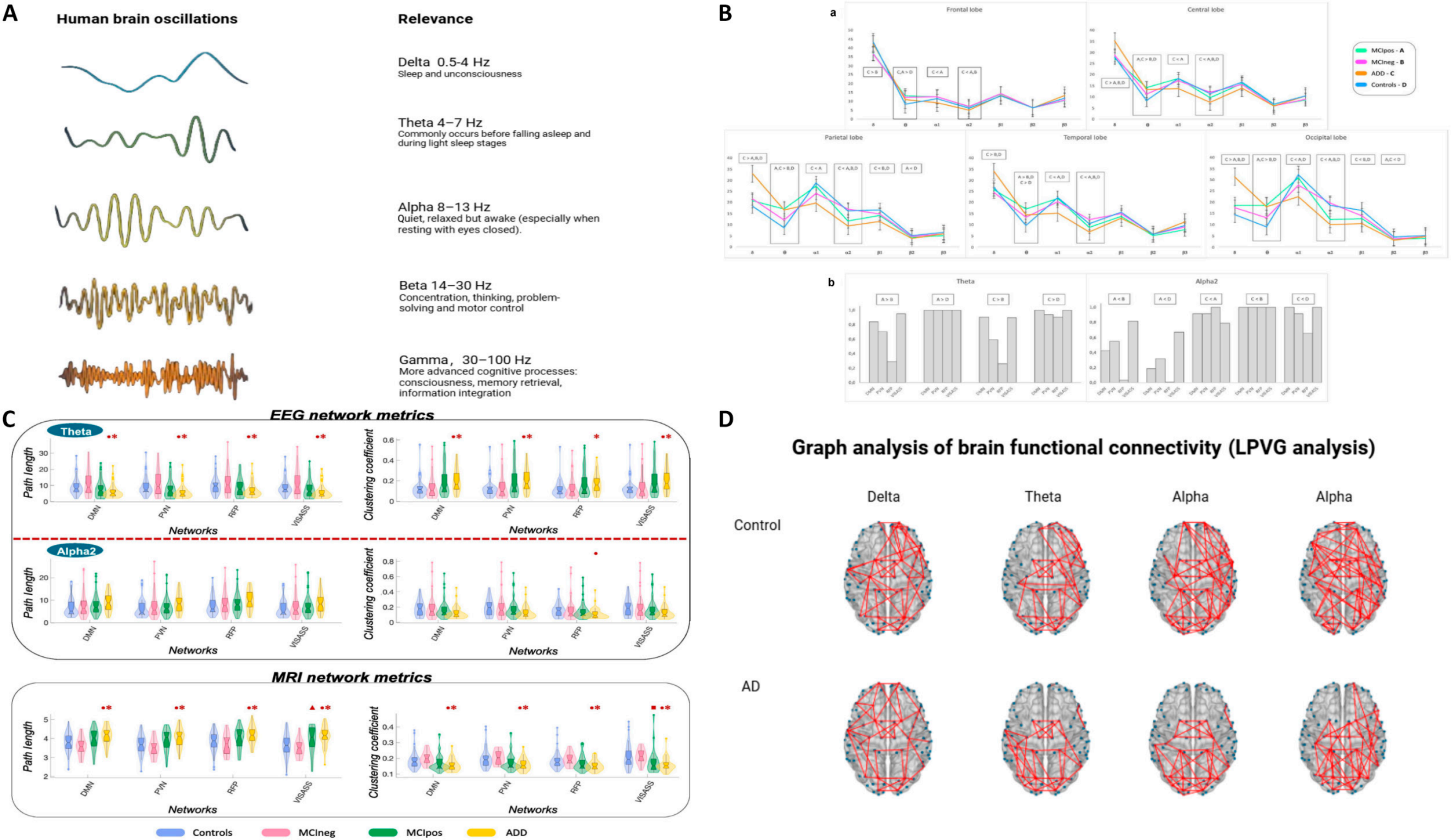
EEG features used for the diagnosis of early-stage AD. Panels (A) and (D) were created with BioRender.com; panels (B) and (C) are adapted from [120]. Copyright © 2021 Elsevier B.V. Licensed under a CC BY-NC-ND 4.0 license. This material has not been altered in any way (including format conversion, which does not constitute a derivative work). (A) Common EEG waveform characteristics that hold clinical significance and are frequently used in the diagnostic assessment of various neurological disorders; (B) Lobar (a) and network (b) current source density analysis on EEG data. (a) Mean values of lobar current density at the different frequencies are reported for MCIpos (A), MCIneg (B), ADD (C), and controls (D). Significant comparisons reported in the boxes are referred to age-, sex- and education-adjusted analysis of variance (ANOVA) models of rank-transformed values, followed by post hoc pairwise comparisons (Bonferroni-corrected for multiple comparisons, *P* < 0.05). Error bars are shown. Lengthened boxes mark the 2 selected frequencies for the subsequent EEG analysis. (b) Percentage of voxels within each selected RS-fMRI (resting-state functional magnetic resonance imaging) network. Only comparisons showing significant differences in at least one network are reported for each frequency band (*P* < 0.05). *P* value refers to age-, sex-, and education-adjusted ANOVA models (false discovery rate-corrected, *P* < 0.05) of rank-transformed values. ADD, Alzheimer’s disease dementia; DMN, default mode network; MCIneg, mild cognitive impairment with pTau/Aß42 < 0.13; MCIpos, mild cognitive impairment with pTau/Aß42 ≥ 0.13; PVN, primary visual network; RFP, right frontal-parietal network; VISASS, visual-associative network. (C) RS-fMRI and EEG graph analysis properties within fMRI networks. Violin plots of clustering coefficient and path length of selected networks (the 2 most significant metrics) are shown for patient groups and healthy controls. Both graph metrics were calculated based on the linear lagged connectivity for alpha2 and theta frequency bands from RS-EEG data (on the top of the figure) and based on Pearson’s correlation coefficient from RS-fMRI data (on the bottom of the figure). Boxplots are reported within violin plots. The horizontal lines in each box plot represent, from the bottom to the top, the 25th percentile, the median, and the 75th percentile. Whiskers represent the minimum and maximum values. All the dots outside the confidence interval are considered as outliers. Significant comparisons are reported (*P* < 0.05). *P* values refer to age-, sex-, and education-adjusted ANOVA models of rank-transformed values, followed by post hoc pairwise comparisons (Bonferroni-corrected for multiple comparisons). ●, ADD versus controls; *, ADD versus MCIneg; ■, MCIpos versus controls; ▲, MCIpos versus MCIneg. (D) Graph analysis of brain functional connectivity [LPVG (limited penetrable visibility graph) analysis] comparing AD patients and healthy controls shows that, across delta, theta, alpha, and beta frequency bands, AD patients exhibit reduced interregional brain connectivity relative to normal individuals.

Due to the demand for low-cost and high-accuracy diagnostic methods, biomarkers have evolved from traditional PET-MRI, Aβ protein concentration in CSF [48], and tau protein concentration [49] to the use of multimodal combined data [50] and EEG in diagnosing mild cognitive impairment (MCI) and AD [51]. EEG biomarkers identified for these conditions have achieved accuracy rates exceeding 70% in classifying the disease into 3 stages [52]. Researchers have found that, in cross-validation predictive analysis, EEG features outperform CSF + APOE biomarkers in predicting the age of onset and disease progression. The combination of EEG + CSF + APOE measurements provides optimal performance across all prediction targets. EEG markers for AD exhibit distinct characteristics: Early patients show a trend of transitioning to low-frequency rhythms [α wave (8 to 15 Hz) and β wave (16 to 31 Hz) power decrease, while θ wave (4 to 8 Hz) and Δ wave (0.5 to 4 Hz) power increase] (Fig. 2A to C) and the weakening of connections between different brain regions (Fig. 2D), with specific spatial distribution patterns primarily in the mid-frontal and prefrontal regions [53,54]. This indicates spatial specificity. Among BCI signals used to assess patients with AD, P300 and SSVEP (steady-state visual evoked potential) are the most commonly employed. P300 is an event-related potential (ERP) that appears as a prominent positive deflection in the EEG approximately 300 ms after an individual attends to and discriminates a “rare/important stimulus”. The amplitude and latency of P300 are closely associated with cognitive function, making it a key signal for many BCIs, particularly in speller paradigms. Classification algorithms can affect diagnostic sensitivity [55]. Studies have confirmed that individuals with MCI or AD exhibit prolonged P300 latency compared to healthy controls, and treatment with pro-cognitive drugs can modulate P300 parameters [56] [not peer-reviewed; data cited from SSRN preprint platform].

SSVEP is a continuous oscillatory response: When a person fixates on a visual stimulus or pattern flashing at a fixed frequency (e.g., 8 or 15 Hz), the visual cortex generates EEG oscillations at the same frequency. This signal exhibits strong frequency specificity, allowing distinct stimuli at different frequencies to be clearly separated in the EEG. Both the amplitude and phase-locking ability of SSVEP are reduced in patients with AD/MCI, reflecting impairment in the visual-attention network and serving as an objective indicator of cognitive dysfunction [57].

Combining SSVEP and P300 may provide a more effective approach for BCI-based identification of patients with AD. Kasawala and Mouli [58] recently conducted an experimental study introducing a novel dual-stimulus device based on light-emitting diodes (LEDs), designed to integrate SSVEP and P300 paradigms to enhance SSVEP classification accuracy. The system employed LEDs flashing at 4 different frequencies (7, 8, 9, and 10 Hz), corresponding to forward, backward, right, and left commands. When a user was fixated on a specific LED, the visual cortex produced a steady-state response at the corresponding frequency. Within these flashing sequences, “target events” were introduced; when the target LED underwent a specific change attended by the user, a P300 wave was elicited, verifying the user’s attention and selection of the stimulus in addition to the frequency-specific SSVEP response. The proposed hybrid system achieved a classification accuracy of 86.25%, substantially exceeding the conventional 70% accuracy threshold typically used in BCI system evaluation protocols.

The EBCI offers a solution to these limitations. With its compact, portable design and superior signal acquisition capabilities compared to noninvasive EEG methods, the EBCI can provide long-term, high-quality signal recordings. This advancement promises to substantially enhance the accuracy of AD diagnosis through neural signal biomarkers.

### Potential role of EBCI in the diagnosis and monitoring of AD

In the field of AD monitoring and diagnosis based on electrophysiological biomarkers, there is still a lack of solid clinical evidence indicating that EBCI provides a substantial incremental diagnostic value over conventional EEG sufficient to offset the latter’s noninvasive advantage. Therefore, EBCI cannot yet serve as a general tool for the early diagnosis of AD. Nevertheless, its strengths in long-term dynamic monitoring and deep brain signal acquisition confer unique complementary value in 2 specific patient populations:

1. Patients with uncertain MCI diagnoses who cannot undergo CSF examination: Through a minimally invasive endovascular approach, EBCI enables dynamic monitoring of electrophysiological biomarkers (e.g., theta/alpha power ratio) over a 1- to 3-month period, which helps distinguish AD-related MCI from non-AD MCI.
2. Patients with early AD receiving anti-Aβ antibodies: Once implanted, EBCI allows noninvasive monthly acquisition of electrophysiological signals adjacent to the fornix, allowing direct functional assessments of treatment efficacy. This capability fills a critical gap left by conventional pathological biomarkers (e.g., PET-Aβ), which cannot reflect real-time functional changes.

Compared to scalp EEG, EBCI offers several unparalleled advantages.

First, scalp EEG cannot detect deep brain activity at therapeutic targets crucial to the treatment of AD, leading to a “monitoring blind spot”. The core mechanisms of AD-targeted therapies (e.g., anti-Aβ antibodies) extend beyond Aβ clearance to the restoration of deep memory-related neural networks, such as the fornix–hippocampal pathway and the nucleus basalis of Meynert–cortical projections. Since these targets are located deep in the brain, scalp EEG signals are substantially attenuated and cannot effectively capture local field potentials (LFPs). In contrast, via EBCI, electrodes can be placed within 1 to 2 mm of these targets through a vascular route [59], allowing direct LFP recording.

Second, scalp EEG provides only static, snapshot-like monitoring that fails to capture gradual or fluctuating therapeutic effects. The response to AD-targeted interventions (e.g., anti-Aβ antibodies or DBS) typically exhibits progressive and oscillatory patterns. In particular, subtle improvements in network function, such as a gradual reduction in the theta/alpha ratio, may emerge within 1 to 3 months after treatment, followed by a plateau phase around 6 months after treatment [60]. During this period, transient fluctuations may also occur due to dosage adjustments or comorbidities. Thus, conventional EEG follow-up every 3 months offers only intermittent snapshots, missing critical temporal dynamics. Moreover, poor compliance due to cognitive decline, logistical challenges in repeated hospital visits, and the high cost of several EEG sessions further limit its application. In contrast, once implanted, EBCI enables continuous home-based monitoring via wireless telemetry, markedly improving data continuity and patient adherence.

Clinical studies have shown that pathological improvement and cognitive improvement are not fully synchronized in patients with AD, and approximately 30% of patients show PET-Aβ reduction without measurable cognitive benefit [47]. The core reason lies in the failure of pathological clearance to translate into functional recovery of key neural circuits. Neither scalp EEG nor current monitoring methods can detect this functional–pathological dissociation. EBCI, however, enables target-specific electrophysiological monitoring, determining whether pathological improvements have been effectively converted into functional gain, thereby reducing the risk of overtreatment or ineffective treatment.

### The application of DBS in the treatment of AD patients

In AD patients, in addition to cortical dysfunction indicated by abnormal α oscillations, several functional network disruption patterns affect theta and alpha frequency bands. These patterns are associated with the severity of cognitive impairment or the APOE genotype and may serve as neurophysiological or phenotypic biomarkers of AD. Modulating these nonlinear connectivity patterns could not only aid in early disease detection but also contribute to cognitive improvement in AD patients. For example, the low connectivity pattern of theta waves forms the basis for potential cognitive enhancements in AD patients [61,62]. Moreover, modulating EEG frequencies through specific brain stimulation techniques [63] or targeting particular ion channels [64] in patients with AD represents a highly promising approach in the treatment of AD.

DBS is a neurosurgical procedure that involves implanting electrodes into specific brain regions to send electrical pulses at targeted frequencies. This stimulation is designed to modulate abnormal brain activity, aiming to improve or treat various disorders. DBS is already a standard treatment for Parkinson’s disease, essential tremor, and dystonia and is actively being researched for its therapeutic potential in conditions associated with dysfunctional neural circuits, including major depressive disorder and AD. While current pharmacological treatments for AD typically alleviate symptoms by modulating neurotransmission, they do not alter the disease’s progression. Anatomically guided DBS, on the other hand, offers a more personalized approach by normalizing brain function through the modulation of regional activity. For instance, stimulating γ-frequency oscillations has shown potential for reducing Aβ deposition and improving cognitive outcomes in AD patients [63].

Researchers have identified specific brain targets for directional DBS that show promising therapeutic effects in AD. Among these targets, the fornix has demonstrated the most substantial effects [65]. The fornix, a large bundle of axons located near the midline of the brain’s white matter, connects various nodes of the limbic circuit, controlling the primary pathways for information entering and exiting the hippocampus and medial temporal lobes. It plays a crucial role in cognitive and episodic memory recall [66]. For example, during awake surgery with stimulation at 3 V or higher amplitudes, a 50-year-old morbidly obese male reported vivid recollections of autobiographical events from over 20 years ago. This suggests that fornix stimulation modulates limbic activity and improves memory functions [67]. Additionally, stimulation at the fornix has been shown to increase glucose metabolism in the temporal and parietal regions, which contrasts with the progression of AD [44,68,69]. This was demonstrated in both phase I (*n* = 6) [44] and phase II (*n* = 42) studies [42], which confirmed the safety and effectiveness of DBS. In the phase I trial, we conducted 12-month continuous stimulation DBS on 6 mild AD patients who were receiving continuous medication treatment. The study focused on 3 aspects, including (a) using standardized low-resolution electromagnetic tomography to map the brain regions that are regulated by stimulation and have physiological functions, (b) evaluating whether DBS can use PET to correct the regional changes in AD brain glucose metabolism, (c) using clinical scales and instruments to measure the impact of DBS on cognitive function over time. The results showed that DBS drives neural activity in the memory circuit, including the entorhinal and hippocampal regions, and activates the default mode network of the brain. PET scans revealed that after continuous stimulation for 12 months, there was an early and substantial reversal of glucose utilization in the temporal and parietal lobes. The assessment of the AD Assessment Scale Cognitive Subscale and the Mini-Mental State Examination indicated that the rate of cognitive decline in some patients might improve and/or slow down at 6 and 12 months. There were no serious adverse events. In the phase II trial, 42 patients with mild AD underwent continuous 12-month DBS stimulation. Although 7.1% of the cases experienced severe adverse events [95% confidence interval (CI), 1.5% to 19.5%], it was found that the rate of cognitive decline in patients over 65 years old slowed down within 1 year, which was largely consistent with the results of the initial study.

The mechanisms behind fornix DBS are not fully understood, but research has suggested that DBS may have neurotrophic effects. Modulating the nonlinear connectivity patterns of neural signals in patients with AD not only facilitates early disease detection but also may contribute to cognitive improvement. For instance, reduced connectivity in the theta band has been identified as a potential foundation for cognitive enhancement in AD patients [61,62].

These effects include the increased delivery of endogenous neurotrophic factors, enhanced synaptic protein expression, and the stimulation of neurogenesis. While phase I and II trials have validated the effectiveness and tolerability of DBS, its invasive and expensive nature severely limits its widespread clinical use. Consequently, the development of less invasive therapies, such as the EBCI, is a promising future direction for DBS technology.

### EBCI for DBS in AD

The most commonly implanted vessel in EBCI research is the sheep SSS, a 1- to 2-mm-diameter vessel covering the motor cortex. The SSS is chosen not only because it is located between the inner and outer dura mater, providing an ideal hollow space [70], but also because it offers an anatomical and geometric alternative to many cortical blood vessels near existing neural stimulation targets [33,71,72]. For AD patients, attention should be directed to intracerebral veins with diameters ranging from 0.4 to 1.4 mm. These veins provide pathways to the thalamic fornix and anterior nucleus, both of which are relevant in the treatment of epilepsy and AD [59,73].

Some conceptual validation studies for endovascular electrode arrays have been conducted. Opie et al. [74] assessed the effects of stents and cortical arrays on cortical stimulation in 25 sheep. They used a nickel–titanium alloy stent with platinum electrodes, which was implanted for at least 4 weeks to ensure better integration with the endothelium. Stimulation of facial muscles and limbs was performed, and the results indicated that there were no substantial differences between stent electrode stimulation and cortical array stimulation, suggesting that stent electrode arrays are a viable minimally invasive stimulation strategy.

Similarly, Liu et al. [75] performed endovascular stimulation of the femoral nerve in sheep using stent-mounted electrode arrays. The researchers found that the electromyography (EMG) threshold current for stent-mounted electrodes was within the range of 0.70 to 1.45, indicating that tissue damage from stimulation via endovascular arrays is unlikely. However, since the current required for functional stimulation exceeded the threshold, they suggested incorporating larger electrodes with greater surface areas to limit charge density and allow higher stimulation intensities while ensuring safety for the subjects.

Researchers have also employed computational methods to identify potential stimulation targets within the body for the EBCI, focusing on 17 key areas relevant to clinical conditions such as obsessive–compulsive disorder [59], epilepsy, and AD. Among these targets, the fornix is considered the most feasible for endovascular stimulation. Simulations of endovascular DBS at the fornix demonstrated that when stimulation amplitudes were kept below 5 V, activation of bilateral neurons at the stimulation site was comparable to that achieved by traditional stereotactic DBS [15]. Blood vessels, being highly conductive, exhibit low impedance at their walls, which enables a reduction in the voltage required for stimulation and enhances efficiency. This makes the EBCI an attractive option for DBS in AD patients, as it reduces potential damage to surrounding brain regions and minimizes invasiveness, which is particularly beneficial for elderly patients.

However, the precise placement of the electrode array within the endovascular system is critical for effective stimulation. Our findings indicate that a deviation of just 1 mm from the optimal electrode placement reduces the predicted neuronal activation of the target area by 1% to 10% across the entire stimulation amplitude range. A 5-mm deviation can lead to a 30% to 60% reduction in neuronal activation [59]. To enhance stimulation precision, the study proposed a solution to this challenge. When designing ring-shaped electrodes (such as stent-based electrodes), the structure was divided into multiple electrically isolated contacts rather than a single continuous electrode. Through software control, the stimulation configuration, including the cathode–anode pairing and current amplitude of each contact, can be adjusted dynamically. This approach enables fine-tuning of the electric field distribution without physically repositioning the electrode, thereby compensating for field variations caused by minor electrode displacements or tissue encapsulation [59]. This also highlights the importance of precise placement during the surgical procedure, and it is expected that future advancements in this technology, in combination with stereotactic techniques, will further improve clinical outcomes.

To achieve precise implantation of endovascular electrodes, one of the major challenges lies in accurately navigating catheters through the complex intracranial vascular network. Recent advances in endovascular navigation technologies have offered several promising solutions to this issue. Magnetically steered catheters enable directional control of the catheter tip via externally applied magnetic fields. These devices exhibit excellent flexibility and remote operability, and have demonstrated the potential to enhance navigation accuracy in various interventional procedures [76]. However, their widespread adoption in delicate electrode implantation is limited by high system complexity, elevated costs, and relatively constrained responsiveness of the catheter to magnetic control.

An alternative and increasingly promising approach involves the integration of AI-driven real-time imaging with high-resolution modalities such as intravascular optical coherence tomography (OCT). OCT offers micrometer-level resolution of vascular wall structures, facilitating the identification of fine anatomical landmarks at the intended implantation sites [77]. When paired with AI algorithms for image segmentation and feature recognition, these systems can provide automated navigation cues, assisting surgeons with highly accurate localization while improving procedural safety and efficiency [78].

Going a step further, the combination of magnetic steering and AI-guided imaging presents the opportunity to develop an integrated system that merges coarse trajectory planning with fine-scale navigational precision. This “macro-to-micro” guidance strategy holds substantial promise for improving the accuracy, reproducibility, and safety of endovascular electrode placement. As such, it represents a forward-looking direction in the evolution of neurointerventional navigation technologies.

### The role of closed-loop control in EBCI for AD treatment

**Fig. 3.**
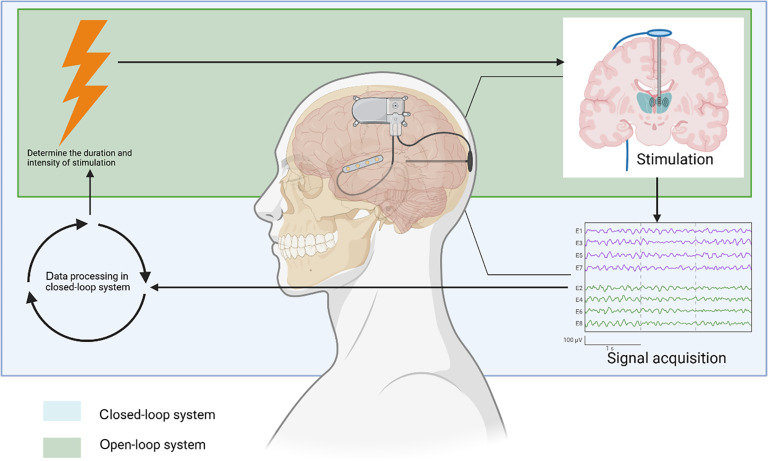
Difference between closed-loop and open-loop DBS systems. The green box contains the open-loop DBS process, and the blue box contains the closed-loop DBS process. Created in BioRender.

A key improvement needed for the development of EBCI technology in the treatment of AD is the integration of a closed-loop system [79]. Currently, most deep brain modulation interfaces (DBMIs) operate using an open-loop system, such as the one employed in traditional DBS treatments. In this system, stimulation parameters like intensity, frequency, and duration are pre-set and remain unchanged throughout the treatment, irrespective of the patient’s evolving brain activity or pathology [80,81] (Fig. 3). However, as brain function and disease states are inherently dynamic, nonspecific stimulation patterns may lead to the development of “tolerance” and reduced modulation effectiveness, potentially disrupting normal brain function and causing side effects [29,82].

Research on closed-loop DBS for epilepsy patients has demonstrated that stimulation can improve memory during low-coding states, but can be detrimental during high-coding states [83]. Closed-loop stimulation has proven more effective than open-loop VNS (vagus nerve stimulation) in reducing both the frequency and severity of seizures [84,85].

In AD, closed-loop systems offer the potential for more personalized treatment. For instance, research in rats has shown that synaptic correlates of memory, such as long-term potentiation (LTP), are highly sensitive to precisely timed electrical stimulation [86]. This suggests that continuous high-frequency pulses, as currently used in DBS for AD patients, may exacerbate symptoms in some cases, explaining why some patients fail to experience the expected benefits from DBS [42,43]. In AD research, hippocampal theta activity has been shown to be a reliable marker for closed-loop DBS, and this technology has already been implemented successfully in sheep models. Moreover, hippocampal gamma waves and phase–amplitude coupling (PAC) are promising markers for closed-loop control in AD treatment [87–90]. As neuro-signal-based diagnostics continue to advance, AD will benefit from more precise and sensitive electronic biomarkers, enabling tailored closed-loop control for both invasive BCI and EBCI.

## Application of EBCI in Neurofeedback Training for AD Patients

EBCI has made substantial strides in the field of neurofeedback, showing promising results in clinical trials. In a pioneering human clinical trial, 4 male participants with upper limb paralysis in Australia who received EBCI treatment were able to independently operate a laptop and perform various daily activities after the implantation of EBCI [14]. None of the participants experienced long-term neurological effects or serious adverse events such as vascular occlusion or thrombosis. Throughout the entire study, the EBCI maintained a stable signal strength, enabling the participants to successfully control the computer to send emails, send text messages, conduct online banking transactions, and make purchases.

However, replicating such results in AD patients presents a unique challenge. The application of BCIs in neurofeedback for AD patients has proven difficult [91,92], primarily because BCIs are traditionally designed to require a fully intact cognitive system to function effectively as a communication tool [93]. Additionally, most currently developed BCIs demand active user participation and prolonged training to learn self-regulation of brain activity [94]. The cognitive decline experienced by AD patients hinders their ability to effectively use these devices.

For a long time, neurofeedback using BCIs was primarily focused on patients with paralysis and other neurodegenerative diseases. Some researchers have attempted to use neurofeedback to improve attention in AD patients by using RSVP (rapid serial visual presentation) keyboards, a type of BCIs that does not rely on a matrix layout [95] [not peer-reviewed; data cited from 2012 IEEE ICASSP conference proceeding]. Unlike the commonly used P300-based spellers, RSVP keyboards do not require stable and accurate gaze fixation, making them more suitable for AD patients who struggle with maintaining attention for extended periods. Despite the initial hopes that neurofeedback would help AD patients improve their performance with RSVP keyboards, the results have been disappointing [96].

Maybe the RSVP keyboard paradigm is overly complex for patients with AD. Therefore, researchers have proposed a simpler and more effective training paradigm for patients with AD: EEG electrodes (Fz, Cz, Pz) are used to record the ratio of specific frequency bands, namely, θ/α. Theta waves (4 to 7 Hz) are typically associated with drowsiness, reduced attention, and memory-encoding deficits, and are often excessively elevated in elderly individuals or patients with dementia. Alpha waves (8 to 12 Hz) are related to relaxation, awake attention, and information integration. In healthy individuals, alpha activity remains relatively stable, but it diminishes with cognitive impairment. Therefore, reducing the θ/α ratio (either by decreasing theta or increasing alpha) is considered beneficial for cognitive function.

During training, patients sit in front of a screen and imagine specific scenarios under the guidance of researchers. They learn to regulate brain activity through feedback signals, such as progress bars, animations, or audio cues. When the EEG device detects increased alpha or decreased theta activity, the system provides reward feedback, such as increased brightness, clarity, or volume of a video. Conversely, a decrease in these stimuli occurs when the desired brain activity is not achieved. This approach aims to induce self-directed improvement in attentional control. Surmeli et al. [97] applied this paradigm to 10 patients with AD meeting the NINCDS-ADRDA (National Institute of Neurological and Communicative Disorders and Stroke–Alzheimer’s Disease and Related Disorders Association) criteria, who were trained twice weekly for 30 sessions. Cognitive function was assessed before and after training using the Cambridge Cognitive Examination (CAMCOG). Over the course of training, the cognitive level of patients with AD did not decline. In fact, the average of CAMCOG total scores increased by 2%, with particularly notable improvements in memory and learning subdomains.

Building on this, Surmeli et al. proposed an individualized EEG-neurofeedback (EEG-NFB) training design. Unlike previous EEG-NFB studies, they quantitatively analyzed each patient’s EEG patterns, compared them to a healthy population database, and identified abnormal frequency bands and brain regions. Instead of training a fixed frequency band, subsequent training targeted the frequency bands and regions most needed for modulation in each patient. This personalized approach allowed for more targeted intervention and could accommodate several types of dementia, including AD and vascular dementia (VD) [97]. Comparisons of the mini-mental state examination (MMSE) scores and EEG signals before and after training revealed improvements in neural function. These studies collectively suggest that BCI-based neurofeedback therapy can effectively enhance cognitive abilities in patients with AD.

Despite cognitive decline, AD patients retain the ability to express emotions (e.g., happiness or sadness) and certain cognitive states (e.g., “yes” or “no” thinking). Some researchers have proposed a paradigm shift from tool-based learning to classical conditioning, introducing an “emotional brain–computer interface” [94]. The core concept of this BCI approach is to omit the active learning component of the paradigm, making it suitable for patients with AD with more severe cognitive impairment who cannot learn the conventional paradigm. Instead, the system uses 2 highly contrasting emotional cues, laughter and crying, to substitute for positive and negative feedback. In healthy participants, classifiers have already successfully distinguished brain activity patterns corresponding to YES versus NO thoughts. This suggests that, in theory, even patients with AD suffering from severe cognitive deficits can use conditioned YES/NO signals to achieve basic communication in the future. This model analyzes BOLD signals (blood oxygen level-dependent signals) to detect basic emotions or cognitive states, thus providing a communication channel for individuals with cognitive impairments. However, this model depends on the collection of BOLD signals, and the use of functional MRI (fMRI)-based BCIs can interfere with EEG signal quality. The MRI scanner’s magnetic field can disrupt the EEG system, leading to reduced signal quality in noninvasive EEG acquisition.

EBCI, combined with functional NIRS (fNIRS), may mitigate the discomfort caused by the prolonged noise associated with fMRI technology. Moreover, by providing higher-quality signal acquisition and more comprehensive coverage of brain regions, EBCI offers greater potential for neurofeedback applications. By integrating fNIRS and EBCI, it is expected that the quality of EEG signals could be improved, offering a more reliable method for neurofeedback in AD patients. This combination of technologies could enhance the effectiveness of BCIs for neurofeedback training, making them a more viable option for AD treatment.

Therefore, we boldly propose a future therapeutic outlook for patients with AD using EBCI: Potential patients can undergo early diagnosis through EBCI, and once confirmed, low-risk neurofeedback interventions utilizing superior, high-quality signals can be implemented in the early stages to enhance attention and focus. For patients with severe pathological features, treatment can be combined with endovascular DBS to address both pathological abnormalities and clinical symptoms. Furthermore, neurofeedback paradigms aimed at improving attention should not be limited to visual and auditory feedback. Incorporation of appropriately calibrated DBS currents can provide functional feedback, creating a trimodal, integrative therapeutic approach.

### Prospects for the application of EBCI in the rehabilitation of patients with AD

To clarify the clinical positioning of EBCI in the treatment of AD, we conducted a comparative analysis across 4 key dimensions: invasiveness, targeting precision, core mechanism of action, and associated risks and limitations. EBCI was compared with conventional DBS, noninvasive 40-Hz sensory entrainment stimulation, transcranial focused ultrasound (tFUS), and next-generation EEG/fNIRS-based neurofeedback technologies. The detailed comparison is summarized in Table 4.

**Table 4.** Comparative characteristics of EBCI and other neuromodulation/neurofeedback approaches for AD intervention

Technique	Invasiveness	Targeting precision	Core mechanisms of action	Risks and limitations
EBCI	Minimally invasive (endovascular implantation without craniotomy)	Millimeter-level (dependent on vascular anatomy; capable of reaching deep nuclei and white matter tracts)	1. Targeted modulation of memory-related circuits (e.g., fornix and nucleus basalis of Meynert) 2. Acquisition of high-fidelity neural signals for neurofeedback training	Vascular injury (<3%), thrombosis (0–2%), mild inflammatory response
Conventional DBS	Highly invasive (craniotomy with stereotactic electrode implantation)	Submillimeter-level (direct neuronal contact)	1. Regulation of neural circuit excitability 2. Promotion of neurotrophic factor release 3. Enhancement of cerebral metabolism	Significant surgical trauma; intracranial hemorrhage (3–5%), infection (2.6–10%), cognitive complications (15–20%)
Noninvasive 40-Hz sensory entrainment stimulation	Noninvasive (visual or auditory stimulation)	Low (diffuse whole-brain activation)	Induction of synchronized γ oscillations	Visual fatigue; variability in auditory tolerance; limited cognitive improvement in mild-to-moderate AD during phase I clinical trials
tFUS (transcranial focused ultrasound)	Noninvasive (ultrasound penetrating the skull)	Moderate (centimeter-level focus; limited precision for deep targets)	1. Reversible opening of the blood–brain barrier (facilitating drug delivery) 2. Local neuromodulation	Risk of thermal injury to brain tissue; variations in skull thickness and tissue density reduce targeting accuracy
Next-generation EEG/fNIRS systems	Noninvasive (integrated multimodal acquisition cap)	Low (restricted to cortical surface signals; deep brain activity attenuated)	1. Neurofeedback training via modulation of functional brain networks 2. Identification of multimodal biomarkers combining fNIRS and EEG	Signal interference from scalp oil or motion artifacts; discomfort during prolonged use; limited to superficial cortical recording; inability to stimulate deep targets

As illustrated in Table 4, EBCI is minimally invasive but offers highly targeted deep brain signal acquisition and precise stimulation. As a rehabilitation tool, this core feature provides potential advantages in the following clinical scenarios.

Early intervention in patients with mild AD: In this population, cognitive function has not yet markedly declined, but conventional DBS carries excessive surgical risk, and noninvasive techniques lack sufficient targeting precision. EBCI precisely stimulates the fornix and nucleus basalis of Meynert via endovascular access, combined with neurofeedback training to enhance attention and memory encoding, thereby potentially decelerating disease progression. This approach is particularly suitable for elderly patients with comorbidities, such as hypertension or diabetes, who cannot tolerate open-cranial procedures.

Patients intolerant to or unresponsive to conventional DBS: EBCI may serve as an alternative for individuals suffering from cognitive complications (e.g., executive dysfunction) or electrode displacement following traditional DBS. Endovascular implantation prevents cortical injury, while the closed-loop system can dynamically adapt to changes in brain networks. Moreover, the stability of stent-based electrodes (demonstrated to remain in place for over 190 d) addresses the long-term decrease in efficacy commonly observed with conventional DBS.

Long-term closed-loop modulation in patients with moderate-to-severe AD: In this group, brain network dysfunction progresses dynamically, making conventional open-loop stimulation difficult to adapt. EBCI allows real-time monitoring of hippocampo-fornical θ–γ coupling and Aβ-related electrophysiological features, enabling dynamic adjustment of stimulation parameters. Concurrently, neurofeedback can help preserve residual cognitive function, filling a critical gap in effective neuromodulation strategies for moderate-to-severe AD.

Based on these considerations, we proposed a future therapeutic blueprint for patients with AD: In the diagnostic domain, EBCI leverages its advantages in long-term dynamic monitoring and deep brain functional capture to provide complementary diagnostic insights and high-quality target-specific signals for neurofeedback rehabilitation. In the stimulation domain, its high targeting precision and capacity for closed-loop modulation can concurrently address pathological abnormalities and clinical symptoms, establishing an integrated “3-in-1” therapeutic strategy.

### Summary of key findings and supporting evidence

Given that EBCI is an emerging technology with limited clinical studies and long-term application data, it is important not to overstate its potential or mislead readers. Table 5 summarizes the core points presented in this article, clearly distinguishing between capabilities that have been empirically validated and those that remain inferential or hypothetical.

**Table 5.** Core findings of EBCI in the treatment of AD and supporting evidence

Core finding	Supporting evidence	Level of realization
1. EBCI enables precise stimulation of key AD targets (fornix and nucleus basalis of Meynert) via endovascular access	Validation of stent-based electrode implantation adjacent to the fornix in sheep models; vascular anatomical analysis in AD patients; clinical efficacy of conventional DBS at the same targets used for inference	Highly likely
2. EBCI signal acquisition performance (LFP/spike recording) can support identification of early AD biomarkers	Stable 12-month signal acquisition in ALS patients; established human applications of AD-related EEG biomarkers (θ/α ratio, P300); 190-d signal stability in sheep models	Possible
3. Minimally invasive nature of EBCI (no craniotomy) reduces surgical risk in AD patients (primarily elderly)	Safety data from human venous sinus stent implantation (bleeding <3%, infection <1%); epidemiological evidence of poor outcomes for elderly patients undergoing craniotomy	Confirmed
4. Closed-loop modulation via EBCI can improve cognitive function in AD patients (e.g., memory and attention)	Neural activation verified in sheep models with closed-loop stimulation; cognitive improvement with conventional DBS in AD is limited; closed-loop DBS in epilepsy demonstrates memory enhancement under low-coding states; preliminary neurofeedback results in AD extrapolated	Hypothetical
5. EBCI has the potential to enhance future applications in the neurofeedback domain.	EEG-based personalized neurofeedback (NFB) protocols and affective brain–computer interface paradigms have demonstrated superior efficacy compared with conventional neurofeedback approaches. Moreover, the demand for individualized rehabilitation strategies is expected to grow substantially in the future. Importantly, patients with moderate-to-severe AD often exhibit highly disorganized cortical EEG activity, making conventional EEG-based neurofeedback difficult to implement and analyze.	Hypothetical
6. EBCIs can be chronically implanted (≥1 year) in patients with AD while maintaining stable functional performance.	Twelve-month implantation data in ALS patients demonstrating safety and signal stability; 190-d long-term recording results in ovine models; and established biocompatibility data from endovascular stent technologies.	Highly likely

## Future Developments in EBCI

### Hardware development of EBCI

Advancements in miniaturization and the need for long-term recordings have led to the evolution of endovascular recording devices from guidewire recordings to microfilament/nanowire recordings, and then to catheter- and stent-based recordings. The most advanced EBCI currently combines a stent with an electrode array. The self-expanding stent securely holds the electrode array in place, facilitating prolonged signal acquisition and therapeutic use.

In current research, the stents are made from nitinol [98], a material known for its superelasticity and biocompatibility. Nitinol forms a passive titanium oxide surface coating that protects it from corrosion, enhancing its stability [99]. In in vivo studies, an animal experiment published in *Biomaterials* demonstrated that surface-modified nitinol stents substantially reduced local nickel ion release and inflammatory responses after implantation, thereby maintaining long-term vascular patency. Taken together, these findings highlight nitinol’s excellent mechanical adaptability to the dynamic vascular environment, along with its favorable biocompatibility and low thrombogenicity in both in vitro and in vivo settings, further reinforcing its potential as a promising material for vascular stent applications [100]. When paired with a platinum electrode array [98,101], the nitinol stent, made from a high-elasticity, biocompatible alloy, can be precisely positioned within blood vessels and transported to the target location using an optimal 4-Fr catheter system [102]. Studies have shown that replacing electrode materials with nitinol improves stability and provides a comparable signal-to-noise ratio to that of stainless steel and platinum [99], which substantially advances the development of long-term intracranial recording via EBCI. Current endovascular stent electrodes are typically connected percutaneously to remote sensing electronic devices located in the subclavian fossa, allowing for signal transmission, storage, and instruction issuing, a method already applied in pacemaker control.

Achieving long-term implantation of EBCI in patients remains a challenge that requires further optimization. Over time, endothelial cells may grow over the electrode surface, a process known as endothelialization. This process is beneficial for long-term implantation, as endothelial coverage of the stent can effectively reduce the risk of thrombosis [103] and decrease noise caused by blood flow, thereby improving signal stability [14]. John et al. [103] implanted an endovascular neural interface (ENI) in the venous sinus of sheep, and after more than 1 month (33 to 54 d), the ENI still clearly recorded penicillin-induced epileptiform spiking activity.

Gerboni et al. reported the stability of neural recordings from stentrode electrodes during the first critical 30 d after implantation [104]. By plotting the impedance magnitude and phase spectra of the intravascular electrodes over time and subtracting the average value from the first recording day, the amplitude changes across frequencies were minimized. During the initial period (~2 weeks after implantation), dynamic changes at the electrode–tissue interface occurred due to endothelialization, causing phase shifts in impedance and slight declines in signal quality with inter-electrode variability [104]. In the stabilization period (2 to 3 weeks after implantation), the electrode–tissue interface achieved preliminary encapsulation, the downward trend in signal quality ceased, inter-electrode consistency substantially improved, and the system entered a long-term, stable recording state [103].

However, endothelialization also introduced new challenges. In cases of device malfunction or upgrade, removing or replacing an EBCI that is tightly integrated with the vessel wall may be difficult and lead to bleeding. Tarigoppula [105] conducted EBCI explantation 7 d after implantation. During removal, neointimal proliferation and mild inflammation were observed at the implantation site, indicating partial endothelialization of the device. Nevertheless, the procedure was successful, with no adverse events, such as bleeding or tissue damage.

For longer-term extractions, Issa [106] evaluated the safety and efficacy of transvenous lead extraction (TLE) in patients having pacemaker or ICD (implantable cardioverter-defibrillator) leads for ≥20 years. Among 124 patients, complete procedural success was achieved in 112/124 (90.3%), and clinical success was achieved in 119/124 (96.0%). Leads implanted for <20 years demonstrated higher clinical success rates (complete plus partial success: 99.2% versus 95.6%, *P* = 0.017). Although this study demonstrated relatively high success rates for long-term intravascular device removal, it did not specifically investigate EBCI. Therefore, further experimental evidence and hardware iteration are needed to fully assess the long-term safety and explantability of EBCI implants.

### Miniaturization and flexible transport of EBCI

The diameter of the intravascular stent and its method of delivery are crucial optimization factors for the development of EBCI, as they determine the device’s ability to reach the target region and its potential for complications. Currently, clinical studies are experimentally targeting the SSS, as the diameter of the current intravascular stent is well-suited to the human venous sinus, which has an average diameter of 2.4 mm. The stiffness of the distal catheter plays a critical role in the delivery process. Larger, stiffer catheters are more prone to causing dissections, while softer catheters are less likely to advance distally. Research indicates that a proximal catheter support technology, which applies forward pressure and torsional delivery, is most effective for overcoming challenging sections, such as the annular segment.

Among the various catheters on the market, the 4-Fr catheter is recommended, as it has the highest success rate (100%) for implanting a stent into the SSS via a 4-Fr 0.044-inch DAC (distal access catheter). Complications observed with 5-Fr and 6-Fr catheter implants have not occurred in clinical studies. In current sheep models, the largest lumen diameter of a catheter that can safely deliver stents to the SSS has an inner diameter of 1.1 mm [102], and stents have been successfully delivered into the sheep venous sinus (with a diameter of 1.59 ± 0.19 mm). The SSS’s anatomical structure suggests that this technique is suitable for smaller veins, such as the 1.4-mm-diameter intracranial veins, where stimulation can be used to treat AD. However, implantation into smaller cortical blood vessels, particularly in the frontal and temporal regions, remains a challenge.

AD, though a widespread amyloid disease in the brain, has a relatively specific spatial distribution of characteristic brain waves. In the classic visual EEG manifestation of AD, substantial differences between mild AD patients and healthy controls are observed in regions like the mid-frontal and anterior cingulate regions. Therefore, miniaturizing the device to cover the necessary brain regions for acquiring neural signals in AD patients is an essential goal.

Zhang et al. [107] developed an ultra-flexible microvascular probe that can be precisely delivered via cervical vasculature to 100-μm-level blood vessels in the rat brain. This device achieved in vivo electrophysiological recordings of LFPs and single-unit spikes in the rat cortex and olfactory bulb. Histological studies showed minimal immune response and long-term stability. While clinical trials for this device have not yet been conducted, it holds promise for exploring small cortical blood vessels in the brain. Additionally, if the delivery of intravascular stents in AD patients can emulate flexible devices, such as polymer-based wires with magnetic heads for flow and magnetic steering navigation, adverse reactions associated with implantation could be substantially reduced [107].

Currently, the stentrode (intravascular stent) requires a connection of recording sensors to electronic devices, with wires running along the vasculature to the subclavian fossa, where the electronic device is placed to collect neural signals from AD patients or to control brain stimulation. This setup carries several risks, including infection, formation of subcutaneous hematomas, challenges with venous injection, and potential blood vessel rupture and bleeding. Studies show that the incidence of infections associated with DBS bioelectronic implants ranges from 2.6% to 10% [108]. However, over time, technological advancements have decreased the incidence of such complications. For example, a 2005 study reported infection and skin erosion rates of up to 50% in patients [109]. In 2014, Kirkfeldt et al. [110] conducted a clinical review showing that the incidence of complications, including pain (0.4% [95% CI, 0.3% to 0.6%]), subcutaneous hematomas (2.3% [95% CI, 1.9% to 2.7%]), and infections (0.8% [95% CI, 0.6% to 1.1%]), had decreased significantly. A similar trend is expected for the development of remote sensor devices for brain applications.

One potential solution to reduce these risks would be integrating the electronic device functions directly into the intravascular stent, eliminating the need for wires inside the body and reducing complications such as skin erosion and hematoma formation. Achieving this would require further miniaturization of the electronic devices to a size that fits within the stent’s existing functionalities, which could take considerable time. However, once realized, this innovation could dramatically improve the safety and efficacy of EBCI.

### Application of EBCI for information analysis in deeper brain

The goals of the BCIs extend far beyond the cortical regions traditionally studied. One of its greatest advantages lies in its ability to reach deep brain areas with minimal invasive intervention. This characteristic is particularly relevant for applications in AD patients. AD, a neurodegenerative disorder affecting the entire brain, is characterized by predictable atrophy of the cortex and hippocampus [111]. In such cases, retaining neural signals from the cortical regions becomes increasingly challenging as these signals may become sparse, limited, and difficult to decode.

Moreover, signals from the cortex that project to the striatum in normal motor circuits often suffer from signal convergence [112], resulting in redundancy and impairing computational efficiency [113,114]. Collecting signals from deeper brain regions could help alleviate these issues. The EBCI has the ability to reach deep brain structures, such as the basal ganglia, through the anterior cerebral artery or other subcortical veins, offering a potential solution by supporting the residual cortical signals [112,115].

For example, the Meynert basal nucleus is a promising target for DBS in AD patients. As early as 1985, reports indicated that DBS successfully improved cortical glucose metabolism in the stimulated hemisphere of AD patients [116]. Subsequent research into DBS targeting the Meynert nucleus has been validated through phase I trials [117,118] and other case studies [119], demonstrating its potential to alleviate clinical symptoms in AD patients. In theory, an EBCI could enable long-term monitoring and electrical stimulation of this region, potentially allowing for closed-loop regulation. This would avoid the damage associated with invasive BCIs used in phase I trials and bypass cortical deficits, enabling better overall control of the stimulation system.

However, these suggestions remain speculative as neither motor neuroprosthetic devices nor recording arrays have been deployed in AD patients or models with motor cortical dysfunction.

## Data Availability

All data supporting the findings of this study are included within the article.

## References

[1] Weller J, Budson A. Current understanding of Alzheimer’s disease diagnosis and treatment. F1000Res. 2018;7. 30135715 10.12688/f1000research.14506.1 PMC6073093

[2] Wolpaw JR, Birbaumer N, McFarland DJ, Pfurtscheller G, Vaughan TM. Brain-computer interfaces for communication and control. Clin Neurophysiol. 2002;113(6):767–791. 12048038 10.1016/s1388-2457(02)00057-3

[3] Willett FR, Avansino DT, Hochberg LR, Henderson JM, Shenoy KV. High-performance brain-to-text communication via handwriting. Nature. 2021;593(7858):249–254. 33981047 10.1038/s41586-021-03506-2 PMC8163299

[4] Collinger JL, Wodlinger B, Downey JE, Wang W, Tyler-Kabara EC, Weber DJ, McMorland AJ, Velliste M, Boninger ML, Schwartz AB. High-performance neuroprosthetic control by an individual with tetraplegia. Lancet. 2013;381(9866):557–564. 23253623 10.1016/S0140-6736(12)61816-9 PMC3641862

[5] Hochberg LR, Serruya MD, Friehs GM, Mukand JA, Saleh M, Caplan AH, Branner A, Chen D, Penn RD, Donoghue JP. Neuronal ensemble control of prosthetic devices by a human with tetraplegia. Nature. 2006;442(7099):164–171. 16838014 10.1038/nature04970

[6] Mridha MF, Das SC, Kabir MM, Lima AA, Islam MR, Watanobe Y. Brain-computer interface: Advancement and challenges. Sensors. 2021;21(17):5746. 34502636 10.3390/s21175746 PMC8433803

[7] Suner S, Fellows MR, Vargas-Irwin C, Nakata GK, Donoghue JP. Reliability of signals from a chronically implanted, silicon-based electrode array in non-human primate primary motor cortex. IEEE Trans Neural Syst Rehabil Eng. 2005;13(4):524–541. 16425835 10.1109/TNSRE.2005.857687

[8] Gold C, Henze DA, Koch C, Buzsáki G. On the origin of the extracellular action potential waveform: A modeling study. J Neurophysiol. 2006;95(5):3113–3128. 16467426 10.1152/jn.00979.2005

[9] Hochberg LR, Bacher D, Jarosiewicz B, Masse NY, Simeral JD, Vogel J, Haddadin S, Liu J, Cash SS, van der Smagt P, et al. Reach and grasp by people with tetraplegia using a neurally controlled robotic arm. Nature. 2012;485(7398):372–375. 22596161 10.1038/nature11076 PMC3640850

[10] Saxena T, Karumbaiah L, Gaupp EA, Patkar R, Patil K, Betancur M, Stanley GB, Bellamkonda RV. The impact of chronic blood-brain barrier breach on intracortical electrode function. Biomaterials. 2013;34(20):4703–4713. 23562053 10.1016/j.biomaterials.2013.03.007

[11] Polikov VS, Tresco PA, Reichert WM. Response of brain tissue to chronically implanted neural electrodes. J Neurosci Methods. 2005;148(1):1–18. 16198003 10.1016/j.jneumeth.2005.08.015

[12] Schultz RL, Willey TJ. The ultrastructure of the sheath around chronically implanted electrodes in brain. J Neurocytol. 1976;5(6):621–642. 1003257 10.1007/BF01181577

[13] Roitbak T, Syková E. Diffusion barriers evoked in the rat cortex by reactive astrogliosis. Glia. 1999;28(1):40–48. 10498821 10.1002/(sici)1098-1136(199910)28:1<40::aid-glia5>3.0.co;2-6

[14] Mitchell P, Lee SCM, Yoo PE, Morokoff A, Sharma RP, Williams DL, MacIsaac C, Howard ME, Irving L, Vrljic I, et al. Assessment of safety of a fully implanted endovascular brain-computer interface for severe paralysis in 4 patients: The Stentrode with Thought-Controlled Digital Switch (SWITCH) study. JAMA Neurol. 2023;80(3):270–278. 36622685 10.1001/jamaneurol.2022.4847 PMC9857731

[15] John SE, Apollo NV, Opie NL, Rind GS, Ronayne SM, May CN, Oxley TJ, Grayden DB. In vivo impedance characterization of cortical recording electrodes shows dependence on electrode location and size. IEEE Trans Biomed Eng. 2019;66(3):675–681. 30004867 10.1109/TBME.2018.2854623

[16] Penn RD, Hilal SK, Michelsen WJ, Goldensohn ES, Driller J. Intravascular intracranial EEG recording. Technical note. J Neurosurg. 1973;38(2):239–243. 4632831 10.3171/jns.1973.38.2.0239

[17] Driller J, Hilal SK, Michelsen WJ, Sollish B, Katz B, Konig W Jr. Development and use of the POD catheter in the cerebral vascular system. Med Res Eng. 1969;8(4):11–16. 5823257

[18] Bower MR, Stead M, Van Gompel JJ, Bower RS, Sulc V, Asirvatham SJ, Worrell GA. Intravenous recording of intracranial, broadband EEG. J Neurosci Methods. 2013;214(1):21–26. 23313850 10.1016/j.jneumeth.2012.12.027 PMC3593671

[19] He BD, Ebrahimi M, Palafox L, Srinivasan L. Signal quality of endovascular electroencephalography. J Neural Eng. 2016;13(1): Article 016016. 26735327 10.1088/1741-2560/13/1/016016

[20] Oxley TJ, Opie NL, John SE, Rind GS, Ronayne SM, Wheeler TL, Judy JW, McDonald AJ, Dornom A, Lovell TJ, et al. Minimally invasive endovascular stent-electrode array for high-fidelity, chronic recordings of cortical neural activity. Nat Biotechnol. 2016;34(3):320–327. 26854476 10.1038/nbt.3428

[21] Opie NL, John SE, Rind GS, Ronayne SM, Grayden DB, Burkitt AN, May CN, O’Brien TJ, Oxley TJ. Chronic impedance spectroscopy of an endovascular stent-electrode array. J Neural Eng. 2016;13(4): Article 046020. 27378157 10.1088/1741-2560/13/4/046020

[22] Forsyth IA, Dunston M, Lombardi G, Rind GS, Ronayne S, Wong YT. Evaluation of a minimally invasive endovascular neural interface for decoding motor activity. Paper presented at: 2019 9th International IEEE/EMBS Conference on Neural Engineering (NER); 2019 March 20–23; San Francisco, CA, USA.

[23] Shen L, Wen B, Chen Z, Wang M, Han L, Shen B, Cao K, Zhang L, Wu J, Kang X. Revolutionizing intrusive neural interfaces: Vascular stent-based neural electrodes. Paper presented at: 2024 12th International Winter Conference on Brain-Computer Interface (BCI); 2024; Gangwon, Republic of Korea.

[24] Synchron’s brain-computer interface now has Nvidia’s AI. https://www.wired.com/story/synchrons-brain-computer-interface-now-has-nvidias-ai

[25] There’s Neuralink—and there’s the mind-reading company that might surpass it. https://www.wired.com/story/synchron-neuralink-competitor-brain-computer-interfaces

[26] Scheltens P, De Strooper B, Kivipelto M, Holstege H, Chételat G, Teunissen CE, Cummings J, van der Flier WM. Alzheimer’s disease. Lancet. 2021;397(10284):1577–1590. 33667416 10.1016/S0140-6736(20)32205-4 PMC8354300

[27] Braak H, Del Tredici K. Reply: The early pathological process in sporadic Alzheimer’s disease. Acta Neuropathol. 2013;126(4):615–618. 23982593 10.1007/s00401-013-1170-1

[28] Kosik KS. Diseases: Study neuron networks to tackle Alzheimer’s. Nature. 2013;503(7474):31–32. 24218661 10.1038/503031a

[29] Hariz MI, Shamsgovara P, Johansson F, Hariz G, Fodstad H. Tolerance and tremor rebound following long-term chronic thalamic stimulation for parkinsonian and essential tremor. Stereotact Funct Neurosurg. 1999;72(2-4):208–218. 10853080 10.1159/000029728

[30] Sastry RA, Pertsch NJ, Tang O, Shao B, Toms SA, Weil RJ. Frailty and outcomes after craniotomy for brain tumor. J Clin Neurosci. 2020;81:95–100. 33222979 10.1016/j.jocn.2020.09.002

[31] Clegg A, Young J, Iliffe S, Rikkert MO, Rockwood K. Frailty in elderly people. Lancet. 2013;381(9868):752–762. 23395245 10.1016/S0140-6736(12)62167-9 PMC4098658

[32] Seicean A, Seicean S, Schiltz NK, Alan N, Jones PK, Neuhauser D, Weil RJ. Short-term outcomes of craniotomy for malignant brain tumors in the elderly. Cancer. 2013;119(5):1058–1064. 23065678 10.1002/cncr.27851

[33] Oxley TJ, Opie NL, Rind GS, Liyanage K, John SE, Ronayne S, McDonald AJ, Dornom A, Lovell TJH, Mitchell PJ, et al. An ovine model of cerebral catheter venography for implantation of an endovascular neural interface. J Neurosurg. 2018;128(4):1020–1027. 28452616 10.3171/2016.11.JNS161754

[34] He Q, Yang Y, Ge P, Li S, Chai X, Luo Z, Zhao J. The brain nebula: Minimally invasive brain-computer interface by endovascular neural recording and stimulation. J Neurointerv Surg. 2024;16(12):1237–1243. 38388478 10.1136/jnis-2023-021296 PMC11671944

[35] Oxley TJ, Yoo PE, Rind GS, Ronayne SM, Lee CMS, Bird C, Hampshire V, Sharma RP, Morokoff A, Williams DL, et al. Motor neuroprosthesis implanted with neurointerventional surgery improves capacity for activities of daily living tasks in severe paralysis: First in-human experience. J Neurointerv Surg. 2021;13(2):102–108. 33115813 10.1136/neurintsurg-2020-016862 PMC7848062

[36] Ahmed RM, Wilkinson M, Parker GD, Thurtell MJ, Macdonald J, McCluskey PJ, Allan R, Dunne V, Hanlon M, Owler BK, et al. Transverse sinus stenting for idiopathic intracranial hypertension: A review of 52 patients and of model predictions. AJNR Am J Neuroradiol. 2011;32(8):1408–1414. 21799038 10.3174/ajnr.A2575 PMC7964366

[37] Townsend RK, Jost A, Amans MR, Hui F, Bender MT, Satti SR, Maurer R, Liu K, Brinjikji W, Fargen KM. Major complications of dural venous sinus stenting for idiopathic intracranial hypertension: Case series and management considerations. J Neurointerv Surg. 2022;14(1). 10.1136/neurintsurg-2021-017361 PMC8722449 33911014

[38] El Naamani K, Abbas R, Tjoumakaris SI, Herial NA, Zarzour H, Schmidt RF, Rosenwasser RH, Jabbour PM, Evans J, Gooch MR. Venous sinus stenting for idiopathic intracranial hypertension patients with functioning ventriculoperitoneal shunts: A case series. Clin Neurol Neurosurg. 2023;233: Article 107894. 37499303 10.1016/j.clineuro.2023.107894

[39] Kirchhof P, Benussi S, Kotecha D, Ahlsson A, Atar D, Casadei B, Castella M, Diener HC, Heidbuchel H, Hendriks J, et al. 2016 ESC guidelines for the management of atrial fibrillation developed in collaboration with EACTS. Eur Heart J. 2016;37(38):2893–2962. 27567408 10.1093/eurheartj/ehw210

[40] Wilson D, Ambler G, Shakeshaft C, Brown MM, Charidimou A, Al-Shahi Salman R, Lip GYH, Cohen H, Banerjee G, Houlden H, et al. Cerebral microbleeds and intracranial haemorrhage risk in patients anticoagulated for atrial fibrillation after acute ischaemic stroke or transient ischaemic attack (CROMIS-2): A multicentre observational cohort study. Lancet Neurol. 2018;17(6):539–547. 29778365 10.1016/S1474-4422(18)30145-5 PMC5956310

[41] Suswati I, Rahayu MAP, Prasetio AD. Managing mental disorders in intracranial hemorrhage (ICH) patients: A case study on the importance of early recognition and intervention. Brain Hemorrhages. 2024;5(1):42–45.

[42] Lozano AM, Fosdick L, Chakravarty MM, Leoutsakos JM, Munro C, Oh E, Drake KE, Lyman CH, Rosenberg PB, Anderson WS, et al. A phase II study of fornix deep brain stimulation in mild Alzheimer’s disease. J Alzheimer’s Dis. 2016;54(2):777–787. 27567810 10.3233/JAD-160017 PMC5026133

[43] Barcia JA, Viloria MA, Yubero R, Sanchez-Sanchez-Rojas L, López A, Strange BA, Cabrera M, Canuet L, Gil P, Nombela C. Directional DBS of the fornix in Alzheimer’s disease achieves long-term benefits: A case report. Front Aging Neurosci. 2022;14: Article 809972. 35431895 10.3389/fnagi.2022.809972 PMC9011335

[44] Laxton AW, Tang-Wai DF, McAndrews MP, Zumsteg D, Wennberg R, Keren R, Wherrett J, Naglie G, Hamani C, Smith GS, et al. A phase I trial of deep brain stimulation of memory circuits in Alzheimer’s disease. Ann Neurol. 2010;68(4):521–534. 20687206 10.1002/ana.22089

[45] Balasa M, Gelpi E, Antonell A, Rey MJ, Sánchez-Valle R, Molinuevo JL, Lladó A. Clinical features and APOE genotype of pathologically proven early-onset Alzheimer disease. Neurology. 2011;76(20):1720–1725. 21576687 10.1212/WNL.0b013e31821a44dd

[46] Murray ME, Graff-Radford NR, Ross OA, Petersen RC, Duara R, Dickson DW. Neuropathologically defined subtypes of Alzheimer’s disease with distinct clinical characteristics: A retrospective study. Lancet Neurol. 2011;10(9):785–796. 21802369 10.1016/S1474-4422(11)70156-9 PMC3175379

[47] Graff-Radford J, Yong KXX, Apostolova LG, Bouwman FH, Carrillo M, Dickerson BC, Rabinovici GD, Schott JM, Jones DT, Murray ME. New insights into atypical Alzheimer’s disease in the era of biomarkers. Lancet Neurol. 2021;20(3):222–234. 33609479 10.1016/S1474-4422(20)30440-3 PMC8056394

[48] Jia J, Ning Y, Chen M, Wang S, Yang H, Li F, Ding J, Li Y, Zhao B, Lyu J, et al. Biomarker changes during 20 years preceding Alzheimer’s disease. N Engl J Med. 2024;390(8):712–722. 38381674 10.1056/NEJMoa2310168

[49] Ossenkoppele R, van der Kant R, Hansson O. Tau biomarkers in Alzheimer’s disease: Towards implementation in clinical practice and trials. Lancet Neurol. 2022;21(8):726–734. 35643092 10.1016/S1474-4422(22)00168-5

[50] Yu X, Sun X, Wei M, Deng S, Zhang Q, Guo T, Shao K, Zhang M, Jiang J, Han Y. Innovative multivariable model combining MRI radiomics and plasma indexes predicts alzheimer’s disease conversion: Evidence from a 2-cohort longitudinal study. Research. 2024;7:0354. 38711474 10.34133/research.0354 PMC11070845

[51] Huang R. Alzheimer’s disease diagnosis based on the EEG analysis. Stud Health Technol Inform. 2023;308:574–582. 38007786 10.3233/SHTI230887

[52] Jiao B, Li R, Zhou H, Qing K, Liu H, Pan H, Lei Y, Fu W, Wang X, Xiao X, et al. Neural biomarker diagnosis and prediction to mild cognitive impairment and Alzheimer’s disease using EEG technology. Alzheimer’s Res Ther. 2023;15(1):32. 36765411 10.1186/s13195-023-01181-1 PMC9912534

[53] Horvath A, Szucs A, Csukly G, Sakovics A, Stefanics G, Kamondi A. EEG and ERP biomarkers of Alzheimer’s disease: A critical review. Front Biosci (Landmark Ed). 2018;23(2):183–220. 28930543 10.2741/4587

[54] Ferreri F, Miraglia F, Vecchio F, Manzo N, Cotelli M, Judica E, Rossini PM. Electroencephalographic hallmarks of Alzheimer’s disease. Int J Psychophysiol. 2022;181:85–94. 36055410 10.1016/j.ijpsycho.2022.08.005

[55] Ravipati Y, Pouratian N, Arnold C, Speier W. Evaluating deep learning performance for P300 neural signal classification. AMIA Annu Symp Proc. 2023;2023:1218–1225. 38222383 PMC10785884

[56] Derkowski W. *The clinical significance of evoked potentials in the diagnosis of mild cognitive impairment and dementia syndromes*. Rochester (NY): SSRN; 2024.

[57] Sridhar S, Manian V. Assessment of cognitive aging using an SSVEP-based brain–computer interface system. Big Data Cogn Comput. 2019;3:29.

[58] Kasawala E, Mouli S. Dual-mode visual system for brain-computer interfaces: Integrating SSVEP and P300 responses. Sensors. 2025;25(6):1802. 40292964 10.3390/s25061802 PMC11946163

[59] Teplitzky BA, Connolly AT, Bajwa JA, Johnson MD. Computational modeling of an endovascular approach to deep brain stimulation. J Neural Eng. 2014;11(2): Article 026011. 24608363 10.1088/1741-2560/11/2/026011

[60] Ferrero J, Williams L, Stella H, Leitermann K, Mikulskis A, O’Gorman J, Sevigny J. First-in-human, double-blind, placebo-controlled, single-dose escalation study of aducanumab (BIIB037) in mild-to-moderate Alzheimer’s disease. Alzheimers Dement. 2016;2(3):169–176. 10.1016/j.trci.2016.06.002 PMC5651340 29067304

[61] Canuet L, Tellado I, Couceiro V, Fraile C, Fernandez-Novoa L, Ishii R, Takeda M, Cacabelos R. Resting-state network disruption and APOE genotype in Alzheimer’s disease: A lagged functional connectivity study. PLOS ONE. 2012;7(9): Article e46289. 23050006 10.1371/journal.pone.0046289 PMC3457973

[62] Canuet L, Pusil S, López ME, Bajo R, Pineda-Pardo J, Cuesta P, Gálvez G, Gaztelu JM, Lourido D, García-Ribas G, et al. Network disruption and cerebrospinal fluid amyloid-beta and phospho-tau levels in mild cognitive impairment. J Neurosci. 2015;35(28):10325–10330. 26180207 10.1523/JNEUROSCI.0704-15.2015 PMC6605340

[63] Iaccarino HF, Singer AC, Martorell AJ, Rudenko A, Gao F, Gillingham TZ, Mathys H, Seo J, Kritskiy O, Abdurrob F, et al. Gamma frequency entrainment attenuates amyloid load and modifies microglia. Nature. 2016;540(7632):230–235. 27929004 10.1038/nature20587 PMC5656389

[64] Chu F, Tan R, Wang X, Zhou X, Ma R, Ma X, Li Y, Liu R, Zhang C, Liu X, et al. Transcranial magneto-acoustic stimulation attenuates synaptic plasticity impairment through the activation of piezo1 in alzheimer’s disease mouse model. Research (Wash D C). 2023;6:0130. 37223482 10.34133/research.0130 PMC10202414

[65] Jakobs M, Lee DJ, Lozano AM. Modifying the progression of Alzheimer’s and Parkinson’s disease with deep brain stimulation. Neuropharmacology. 2020;171: Article 107860. 31765650 10.1016/j.neuropharm.2019.107860

[66] Senova S, Fomenko A, Gondard E, Lozano AM. Anatomy and function of the fornix in the context of its potential as a therapeutic target. J Neurol Neurosurg Psychiatry. 2020;91(5):547–559. 32132227 10.1136/jnnp-2019-322375 PMC7231447

[67] Hamani C, McAndrews MP, Cohn M, Oh M, Zumsteg D, Shapiro CM, Wennberg RA, Lozano AM. Memory enhancement induced by hypothalamic/fornix deep brain stimulation. Ann Neurol. 2008;63(1):119–123. 18232017 10.1002/ana.21295

[68] Fontaine D, Deudon A, Lemaire JJ, Razzouk M, Viau P, Darcourt J, Robert P. Symptomatic treatment of memory decline in Alzheimer’s disease by deep brain stimulation: A feasibility study. J Alzheimer’s Dis. 2013;34(1):315–323. 23168448 10.3233/JAD-121579

[69] Fontaine D, Santucci S. Deep brain stimulation in Alzheimer’s disease. Int Rev Neurobiol. 2021;159:69–87. 34446251 10.1016/bs.irn.2021.06.005

[70] Patchana T, Zampella B, Berry JA, Lawandy S, Sweiss RB. Superior sagittal sinus: A review of the history, surgical considerations, and pathology. Cureus. 2019;11(5): Article e4597. 31309022 10.7759/cureus.4597 PMC6609282

[71] Nowinski WL, Chua BC, Volkau I, Puspitasari F, Marchenko Y, Runge VM, Knopp MV. Simulation and assessment of cerebrovascular damage in deep brain stimulation using a stereotactic atlas of vasculature and structure derived from multiple 3- and 7-tesla scans. J Neurosurg. 2010;113(6):1234–1241. 20345226 10.3171/2010.2.JNS091528

[72] Liyanage KA, Steward C, Moffat BA, Opie NL, Rind GS, John SE, Ronayne S, May CN, O’Brien TJ, Milne ME, et al. Development and implementation of a corriedale ovine brain atlas for use in atlas-based segmentation. PLOS ONE. 2016;11(6): Article e0155974. 27285947 10.1371/journal.pone.0155974 PMC4902240

[73] Lyons MK. Deep brain stimulation: Current and future clinical applications. Mayo Clin Proc. 2011;86(7):662–672. 21646303 10.4065/mcp.2011.0045 PMC3127561

[74] Opie NL, John SE, Rind GS, Ronayne SM, Wong YT, Gerboni G, Yoo PE, Lovell TJH, Scordas TCM, Wilson SL, et al. Focal stimulation of the sheep motor cortex with a chronically implanted minimally invasive electrode array mounted on an endovascular stent. Nat Biomed Eng. 2018;2(12):907–914. 31015727 10.1038/s41551-018-0321-z

[75] Liu J, Grayden DB, Keast JR, Booth LC, May CN, John SE. Feasibility of endovascular stimulation of the femoral nerve using a stent-mounted electrode array. J Neural Eng. 2024;21(3): Article 036034. 10.1088/1741-2552/ad4f16 38776894

[76] Yang Z, Yang L, Zhang M, Wang Q, Yu SCH, Zhang L. Magnetic control of a steerable guidewire under ultrasound guidance using mobile electromagnets. IEEE Robot Autom Lett. 2021;6(2):1280–1287.

[77] Tearney GJ, Waxman S, Shishkov M, Vakoc BJ, Suter MJ, Freilich MI, Desjardins AE, Oh WY, Bartlett LA, Rosenberg M, et al. Three-dimensional coronary artery microscopy by intracoronary optical frequency domain imaging. JACC Cardiovasc Imaging. 2008;1(6):752–761. 19356512 10.1016/j.jcmg.2008.06.007 PMC2852244

[78] Guo Z, Li X, Huang H, Guo N, Li Q. Deep learning-based image segmentation on multimodal medical imaging. IEEE Trans Radiat Plasma Med Sci. 2019;3(2):162–169. 34722958 10.1109/trpms.2018.2890359 PMC8553020

[79] Senova S, Chaillet A, Lozano AM. Fornical closed-loop stimulation for Alzheimer’s disease. Trends Neurosci. 2018;41(7):418–428. 29735372 10.1016/j.tins.2018.03.015

[80] Chang R, Peng J, Chen Y, Liao H, Zhao S, Zou J, Tan S. Deep brain stimulation in drug addiction treatment: Research progress and perspective. Front Psych. 2022;13: Article 858638. 10.3389/fpsyt.2022.858638 PMC9022905 35463506

[81] Chen D, Zhao Z, Shi J, Li S, Xu X, Wu Z, Tang Y, Liu N, Zhou W, Ni C, et al. Harnessing the sensing and stimulation function of deep brain-machine interfaces: A new dawn for overcoming substance use disorders. Transl Psychiatry. 2024;14(1):440. 39419976 10.1038/s41398-024-03156-8 PMC11487193

[82] Yuen J, Kouzani AZ, Berk M, Tye SJ, Rusheen AE, Blaha CD, Bennet KE, Lee KH, Shin H, Kim JH, et al. Deep brain stimulation for addictive disorders-where are we now? Neurotherapeutics. 2022;19(4):1193–1215. 35411483 10.1007/s13311-022-01229-4 PMC9587163

[83] Ezzyat Y, Kragel JE, Burke JF, Levy DF, Lyalenko A, Wanda P, O’Sullivan L, Hurley KB, Busygin S, Pedisich I, et al. Direct brain stimulation modulates encoding states and memory performance in humans. Curr Biol. 2017;27(9):1251–1258. 28434860 10.1016/j.cub.2017.03.028 PMC8506915

[84] Fisher RS, Afra P, Macken M, Minecan DN, Bagić A, Benbadis SR, Helmers SL, Sinha SR, Slater J, Treiman D, et al. Automatic vagus nerve stimulation triggered by ictal tachycardia: Clinical outcomes and device performance--The U.S. E-37 trial. Neuromodulation. 2016;19(2):188–195. 26663671 10.1111/ner.12376 PMC5064739

[85] Hamilton P, Soryal I, Dhahri P, Wimalachandra W, Leat A, Hughes D, Toghill N, Hodson J, Sawlani V, Hayton T, et al. Clinical outcomes of VNS therapy with AspireSR(®) (including cardiac-based seizure detection) at a large complex epilepsy and surgery centre. Seizure. 2018;58:120–126. 29702409 10.1016/j.seizure.2018.03.022

[86] Hyman JM, Wyble BP, Goyal V, Rossi CA, Hasselmo ME. Stimulation in hippocampal region CA1 in behaving rats yields long-term potentiation when delivered to the peak of theta and long-term depression when delivered to the trough. J Neurosci. 2003;23(37):11725–11731. 14684874 10.1523/JNEUROSCI.23-37-11725.2003 PMC6740943

[87] Montgomery SM, Buzsáki G. Gamma oscillations dynamically couple hippocampal CA3 and CA1 regions during memory task performance. Proc Natl Acad Sci USA. 2007;104(36):14495–14500. 17726109 10.1073/pnas.0701826104 PMC1964875

[88] Lisman JE, Idiart MA. Storage of 7 +/- 2 short-term memories in oscillatory subcycles. Science. 1995;267(5203):1512–1515. 7878473 10.1126/science.7878473

[89] Tort AB, Komorowski RW, Manns JR, Kopell NJ, Eichenbaum H. Theta-gamma coupling increases during the learning of item-context associations. Proc Natl Acad Sci USA. 2009;106(49):20942–20947. 19934062 10.1073/pnas.0911331106 PMC2791641

[90] Siegel M, Warden MR, Miller EK. Phase-dependent neuronal coding of objects in short-term memory. Proc Natl Acad Sci USA. 2009;106(50):21341–21346. 19926847 10.1073/pnas.0908193106 PMC2779828

[91] Gregoriou GG, Gotts SJ, Zhou H, Desimone R. High-frequency, long-range coupling between prefrontal and visual cortex during attention. Science. 2009;324(5931):1207–1210. 19478185 10.1126/science.1171402 PMC2849291

[92] Tayebi H, Azadnajafabad S, Maroufi SF, Pour-Rashidi A, Khorasanizadeh M, Faramarzi S, Slavin KV. Applications of brain-computer interfaces in neurodegenerative diseases. Neurosurg Rev. 2023;46(1):131. 37256332 10.1007/s10143-023-02038-9

[93] Cummings JL, Cole G. Alzheimer disease. JAMA. 2002;287(18):2335–2338. 11988038 10.1001/jama.287.18.2335

[94] Liberati G, Dalboni da Rocha JL, van der Heiden L, Raffone A, Birbaumer N, Olivetti Belardinelli M, Sitaram R. Toward a brain-computer interface for Alzheimer’s disease patients by combining classical conditioning and brain state classification. J Alzheimer’s Dis. 2012;31(Suppl 3):S211–S220. 22451316 10.3233/JAD-2012-112129

[95] Orhan U, Hild KE, Erdogmus D, Roark B, Oken B, Fried-Oken M. RSVP keyboard: An EEG based typing interface. Paper presented at: 2012 IEEE International Conference on Acoustics, Speech and Signal Processing (ICASSP); 2012 March 25–30; Kyoto, Japan. 10.1109/ICASSP.2012.6287966 PMC3771530 24500542

[96] Galvin-McLaughlin D, Klee D, Memmott T, Peters B, Wiedrick J, Fried-Oken M, Oken B. Methodology and preliminary data on feasibility of a neurofeedback protocol to improve visual attention to letters in mild Alzheimer’s disease. Contemp Clin Trials Commun. 2022;28: Article 100950. 35754975 10.1016/j.conctc.2022.100950 PMC9228283

[97] Surmeli T, Eralp E, Mustafazade I, Kos H, Özer GE, Surmeli OH. Quantitative EEG neurometric analysis-guided neurofeedback treatment in dementia: 20 cases. How neurometric analysis is important for the treatment of dementia and as a biomarker? Clin EEG Neurosci. 2016;47(2):118–133. 26099949 10.1177/1550059415590750

[98] Duerig T, Pelton A, Stöckel D. An overview of nitinol medical applications. Mater Sci Eng A. 1999;273-275:149–160.

[99] Barrett RD, Bishara SE, Quinn JK. Biodegradation of orthodontic appliances. Part I. Biodegradation of nickel and chromium in vitro. Am J Orthod Dentofacial Orthop. 1993;103(1):8–14. 8422037 10.1016/0889-5406(93)70098-9

[100] Nagaraja S, Sullivan SJL, Stafford PR, Lucas AD, Malkin E. Impact of nitinol stent surface processing on in-vivo nickel release and biological response. Acta Biomater. 2018;72:424–433. 29597023 10.1016/j.actbio.2018.03.036

[101] Cogan SF. Neural stimulation and recording electrodes. Annu Rev Biomed Eng. 2008;10:275–309. 18429704 10.1146/annurev.bioeng.10.061807.160518

[102] Thierry B, Merhi Y, Bilodeau L, Trépanier C, Tabrizian M. Nitinol versus stainless steel stents: Acute thrombogenicity study in an ex vivo porcine model. Biomaterials. 2002;23(14):2997–3005. 12069342 10.1016/s0142-9612(02)00030-3

[103] John SE, Donegan S, Scordas TC, Qi W, Sharma P, Liyanage K, Wilson S, Birchall I, Ooi A, Oxley TJ, et al. Vascular remodeling in sheep implanted with endovascular neural interface. J Neural Eng. 2022;19(5): Article 056043. 10.1088/1741-2552/ac9a77 36240737

[104] Gerboni G, John SE, Rind GS, Ronayne SM, May CN, Oxley TJ, Grayden DB, Opie NL, Wong YT. Visual evoked potentials determine chronic signal quality in a stent-electrode endovascular neural interface. Biomed Phys Eng Express. 2018;4: 055018.

[105] Tarigoppula VSA. Safe retrieval of a stent-based endovascular neural recording array. In: *2023 11th International IEEE/EMBS Conference on Neural Engineering (NER)*. Baltimore (MD): IEEE; 2023. p. 1–4.

[106] Issa ZF. Outcome of transvenous lead extraction of leads older than 20 years. J Cardiovasc Electrophysiol. 2021;32(11):3042–3048. 34453369 10.1111/jce.15229

[107] Zhang A, Mandeville ET, Xu L, Stary CM, Lo EH, Lieber CM. Ultraflexible endovascular probes for brain recording through micrometer-scale vasculature. Science. 2023;381(6655):306–312. 37471542 10.1126/science.adh3916 PMC11412271

[108] Feldmann LK, Neumann WJ, Faust K, Schneider GH, Kühn AA. Risk of infection after deep brain stimulation surgery with externalization and local-field potential recordings: Twelve-year experience from a single institution. Stereotact Funct Neurosurg. 2021;99(6):512–520. 33971662 10.1159/000516150

[109] Mayberg HS, Lozano AM, Voon V, McNeely HE, Seminowicz D, Hamani C, Schwalb JM, Kennedy SH. Deep brain stimulation for treatment-resistant depression. Neuron. 2005;45(5):651–660. 15748841 10.1016/j.neuron.2005.02.014

[110] Kirkfeldt RE, Johansen JB, Nohr EA, Jørgensen OD, Nielsen JC. Complications after cardiac implantable electronic device implantations: An analysis of a complete, nationwide cohort in Denmark. Eur Heart J. 2014;35(18):1186–1194. 24347317 10.1093/eurheartj/eht511 PMC4012708

[111] Pini L, Pievani M, Bocchetta M, Altomare D, Bosco P, Cavedo E, Galluzzi S, Marizzoni M, Frisoni GB. Brain atrophy in Alzheimer’s disease and aging. Ageing Res Rev. 2016;30:25–48. 26827786 10.1016/j.arr.2016.01.002

[112] Nambu A. Somatotopic organization of the primate basal ganglia. Front Neuroanat. 2011;5:26. 21541304 10.3389/fnana.2011.00026 PMC3082737

[113] Kitano H. Towards a theory of biological robustness. Mol Syst Biol. 2007;3:137. 17882156 10.1038/msb4100179 PMC2013924

[114] Barlow H. Redundancy reduction revisited. Network. 2001;12(3):241–253. 11563528

[115] Romanelli P, Esposito V, Schaal DW, Heit G. Somatotopy in the basal ganglia: Experimental and clinical evidence for segregated sensorimotor channels. Brain Res Brain Res Rev. 2005;48(1):112–128. 15708631 10.1016/j.brainresrev.2004.09.008

[116] Turnbull IM, McGeer PL, Beattie L, Calne D, Pate B. Stimulation of the basal nucleus of Meynert in senile dementia of Alzheimer’s type. A preliminary report. Appl Neurophysiol. 1985;48(1-6):216–221. 3915647

[117] Kuhn J, Hardenacke K, Shubina E, Lenartz D, Visser-Vandewalle V, Zilles K, Sturm V, Freund HJ. Deep brain stimulation of the nucleus basalis of meynert in early stage of Alzheimer’s dementia. Brain Stimul. 2015;8(4):838–839. 25991080 10.1016/j.brs.2015.04.002

[118] Kuhn J, Hardenacke K, Lenartz D, Gruendler T, Ullsperger M, Bartsch C, Mai JK, Zilles K, Bauer A, Matusch A, et al. Deep brain stimulation of the nucleus basalis of Meynert in Alzheimer’s dementia. Mol Psychiatry. 2015;20(3):353–360. 24798585 10.1038/mp.2014.32

[119] Zhang W, Liu W, Patel B, Chen Y, Wang K, Yang A, Meng F, Wagle Shukla A, Cen S, Yu J, et al. Case report: Deep brain stimulation of the nucleus basalis of meynert for advanced Alzheimer’s disease. Front Hum Neurosci. 2021;15: Article 645584. 34122027 10.3389/fnhum.2021.645584 PMC8188895

[120] Cecchetti G, Agosta F, Basaia S, Cividini C, Cursi M, Santangelo R, Caso F, Minicucci F, Magnani G, Filippi M. Resting-state electroencephalographic biomarkers of Alzheimer’s disease. NeuroImage Clin. 2021;31: Article 102711. 34098525 10.1016/j.nicl.2021.102711 PMC8185302

